# A Continuous Add‐On Probe Reveals the Nonlinear Enlargement of Mitochondria in Light‐Activated Oncosis

**DOI:** 10.1002/advs.202004566

**Published:** 2021-07-01

**Authors:** Kang‐Nan Wang, Xintian Shao, Zhiqi Tian, Liu‐Yi Liu, Chengying Zhang, Cai‐Ping Tan, Jie Zhang, Peixue Ling, Fei Liu, Qixin Chen, Jiajie Diao, Zong‐Wan Mao

**Affiliations:** ^1^ MOE Key Laboratory of Bioinorganic and Synthetic Chemistry School of Chemistry State Key Laboratory of Oncology in South China Sun Yat‐Sen University Guangzhou 510275 P. R. China; ^2^ Institute of Materia Medica Shandong First Medical University & Shandong Academy of Medical Sciences Jinan 250000 P. R. China; ^3^ Department of Cancer Biology University of Cincinnati College of Medicine Cincinnati 45267 USA; ^4^ Shandong Academy of Pharmaceutical Science Key Laboratory of Biopharmaceuticals Engineering Laboratory of Polysaccharide Drugs National‐Local Joint Engineering Laboratory of Polysaccharide Drugs Jinan 250101 P. R. China; ^5^ Department of Molecular Genetics, Biochemistry, and Microbiology University of Cincinnati College of Medicine Cincinnati 45267 USA; ^6^ School of Pharmaceutical Sciences Shandong University Jinan 250101 P. R. China; ^7^ Advanced Medical Research Institute/Translational Medicine Core Facility of Advanced Medical Research Institute Shandong University Jinan 250101 P. R. China

**Keywords:** nonlinear enlargement of mitochondria, oncosis, continuous add‐on probe, dual‐color imaging, super‐resolution imaging

## Abstract

Oncosis, depending on DNA damage and mitochondrial swelling, is an important approach for treating cancer and other diseases. However, little is known about the behavior of mitochondria during oncosis, due to the lack of probes for in situ visual illumination of the mitochondrial membrane and mtDNA. Herein, a mitochondrial lipid and mtDNA dual‐labeled probe, **MitoMN**, and a continuous add‐on assay, are designed to image the dynamic process of mitochondria in conditions that are unobservable with current mitochondrial probes. Meanwhile, the **MitoMN** can induce oncosis in a light‐activated manner, which results in the enlargement of mitochondria and the death of cancer cells. Using structured illumination microscopy (SIM), **MitoMN**‐stained mitochondria with a dual‐color response reveals, for the first time, how swelled mitochondria interacts and fuses with each other for a nonlinear enlargement to accelerate oncosis into an irreversible stage. With this sign of irreversible oncosis revealed by **MitoMN**, oncosis can be segregated into three stages, including before oncosis, initial oncosis, and accelerated oncosis.

## Graphical abstract

A mitochondrial lipids and mtDNA dual‐labeling probe with continuous add‐on assay is designed to induce and image the nonlinear enlargement of mitochondria during oncosis. Together with super‐resolution imaging, it is revealed how swelled mitochondria interact and fuse with each other for a nonlinear enlargement to accelerate oncosis into an irreversible stage, which can be a new landmark for staging oncosis process.

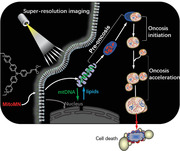

## 1 Introduction

As subcellular organelles with lipid membranes and independent genes (mtDNA), mitochondria play crucial roles in cell proliferation, ATP production, and cellular signaling.^[^ ^1^ ^]^ Mitochondrial processes are not only necessary to maintain cell homeostasis, but also provide energy sources for many biological events, such as cell‐to‐cell communication.^[^ ^2^ ^]^ Likewise, many conditions, including apoptosis, pyroptosis, oncosis, and necrosis, etc., are dependent upon the mitochondrial particular structure damage or the mitochondrial dysfunction.^[^ ^3^ ^]^ One of those conditions may be oncosis, a form of accidental cell death induced by the overload of reactive oxygen species (ROS),^[^ ^4^, ^5^ ^]^ in which abnormal mitochondrial metabolism plays an important role.^[^ ^6^ ^]^ As extent research greatly emphasized, oncosis has been the focus of increasing attention for cancer treatment.^[^ ^7^ ^]^ Although oncosis has been characterized at the cellular level (by cellular swelling, the formation of external vesicles in the plasma membrane, and the presence of dilated organelles),^[^ ^6^ ^]^ the behavior of mitochondria and mitochondrial events during oncosis remains largely unknown. Such knowledge is crucial, however, to understand oncosis and to treat oncosis‐related cancers.

Using fluorescent probes to image subcellular dynamics is one of the feasible solutions for tracking the organelles behavior under physiological/pathological conditions.^[^ ^8^ ^]^ For example, the fluorescent probes, stain the outer mitochondrial membrane based on mitochondrial membrane potential (MMP), have been used to track the dynamic behavior of mitochondria.^[^ ^9^ ^]^ However, fluorescent probes—which can only stain mitochondrial lipids—would not meet the demands for in‐depth understanding of mitochondrial behavior during oncosis, because mtDNA is also likely to be involved in this process.^[^ ^10^ ^]^ Thus, methods of tracking the behavior of mitochondrial membranes and substances within mitochondria, especially mtDNA, remain critically needed. To achieve this goal, an organic molecule that can illuminate both mitochondrial lipids and mtDNA, and simultaneously perform dual‐color response imaging shows outstanding promise. A drawback, however, is that fluorescence microscopy's spatial resolution is limited by light diffraction to only a few hundred nanometers,^[^ ^11^, ^12^ ^]^ which hampers any in situ visual tracking of the mitochondrial membrane and mtDNA. Furthermore, designing single‐molecule fluorescent probes that can provide dual‐color response imaging of mitochondrial lipids and mtDNA presents its own challenges, which not only requires excellent photophysical properties, good mitochondrial imaging capabilities, and low dark cytotoxicity, but also demands a differentiated, selective response to mitochondrial lipids and mtDNA. Indeed, to the best of our knowledge, no literature has reported the dual‐color response imaging of mitochondrial membranes and mtDNA using a single probe.

To date, imaging subcellular dynamics at a high spatial resolution in living cells has generated a wealth of information and, in turn, facilitated significant discoveries.^[^ ^11^, ^13^ ^]^ For instance, structured illumination microscopy (SIM) revealed new, direct, and functional contact forms between the mitochondria and lysosomes, which can increase the local viscosity of mitochondria.^[^ ^14^ ^]^ SIM in living cells can also be used to quantify subcellular dynamics,^[^ ^15^ ^]^ not to mention support drug screening.^[^ ^16^, ^17^ ^]^ Since SIM improves the resolution by using optics and analysis and does not require any nonlinear response of fluorophore to the light intensity,^[^ ^18^ ^]^ SIM has gained popularity in cell biology.^[^ ^19^ ^]^ On the one hand, however, most fluorescent probes cannot meet the requirements of long‐term real‐time dynamic imaging for important biological events. For instance, the photobleaching of fluorescence molecules with laser irradiation can jeopardize their capacity for long‐term imaging. On the other hand, as the mitochondrial surface area expands, the density of fluorescence molecules becomes diluted, which would further decrease the fluorescence intensity.

**Scheme 1:**
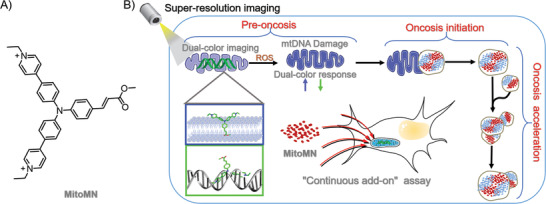
A) The chemical structure of probe MitoMN. B) Schematic representation of nonlinear enlargement of mitochondria in light‐activated oncosis by MitoMN. MitoMN can continuously add‐onto the mitochondria and light‐up the mitochondrial lipids and mtDNA with a clear, dual‐color image. Under irradiation conditions, the ROS produced by **MitoMN** induced DNA damage and mitochondria enlargement, which is accompanied by a dual‐color response in a single organelle. Using super‐resolution imaging, we revealed that the swelled mitochondria interact and fuse with each other for a nonlinear enlargement to accelerate oncosis into an irreversible stage. With this sign of irreversible oncosis revealed by **MitoMN**, the oncosis process can be segregated into three stages, including pre‐oncosis, initial oncosis, and accelerated oncosis.

Inspired by the discussion above, as shown in **Scheme** **1**, a new labeling assay by continuously adding probe molecules onto mitochondria may resolve these technical barriers. The “continuous add‐on” assay could replenish the loosening of fluorescent signals caused by photobleaching and dilution, for long‐term‐dynamic investigation of the mitochondrial dynamics. For the fluorescent probes, considering the structure of the mitochondrial membrane and nucleic acids, the triphenylamine skeleton containing sidearms with different functions provides a great potential for designing the membrane/nucleic acid imaging probes.^[^ ^20^ ^]^ We precisely regulate the sidearm lipophilicity of the triphenylamine skeleton for membrane binding and its positive charges for the weak interaction with nucleic acid, herein, a probe, (Scheme 1) with improved hydrophilicity/hydrophobicity for a better simultaneous lighting‐up of the mitochondrial lipids and mtDNA was developed (named **MitoMN**, which represents mitochondrial membrane and mtDNA dual targeting). Due to the strong binding ability of the **MitoMN** to lipids, the probe will continuously add‐onto the mitochondria, once it was taken up by the cells. In this case, **MitoMN** not only realizes the simultaneous monitoring of mitochondrial lipids and mtDNA, but also satisfies the need for long‐term, real‐time tracking of mitochondria in super‐resolution imaging. Combined with its ability to generate ROS upon light irradiation for inducing cell oncosis, **MitoMN** may be able to induce cancer cell oncosis, and to track the entire oncosis process in situ and in real‐time. By applying super‐resolution nanoscopy, we were able, for the first time, to monitor the process of mitochondrial enlargement to segregate oncosis into three stages: pre‐oncosis, initial oncosis, and accelerated oncosis.

## 2 Results and Discussion

After being synthesized and isolated (Scheme S1, Supporting Information), the probe MitoMN was fully characterized by EMS, ^1^H NMR, and ^13^C NMR (Figures S1–S11, Supporting Information), which revealed the correct structure and high level of purity of the compounds. Results concerning the photophysical properties (Figure S12 and Table S1, Supporting Information) and the density functional theory (DFT) calculations (Figure S13, Supporting Information) confirm that MitoMN is suitable for SIM imaging. The inert fluorescence response of MitoMN in the context of different ROS (i.e., NOO^−^, ClO^−^, O_2_^−^, and H_2_O_2_) indicates that the external ROS would not affect the fluorescence of MitoMN (Figure S14, Supporting Information); MitoMN also presented a stable fluorescence emission in the common bioactive anions and cations in buffer solution (Figure S15, Supporting Information). The MitoMN's superb ability to generate ROS—with a yield of 73.1±0.1%—provided the foundation for efficient oncosis^[^ ^5^ ^]^ (Figure S16, Supporting Information).

### 2.1 Super‐Resolution Characterizes MitoMN in Living Cells

**Figure 1:**
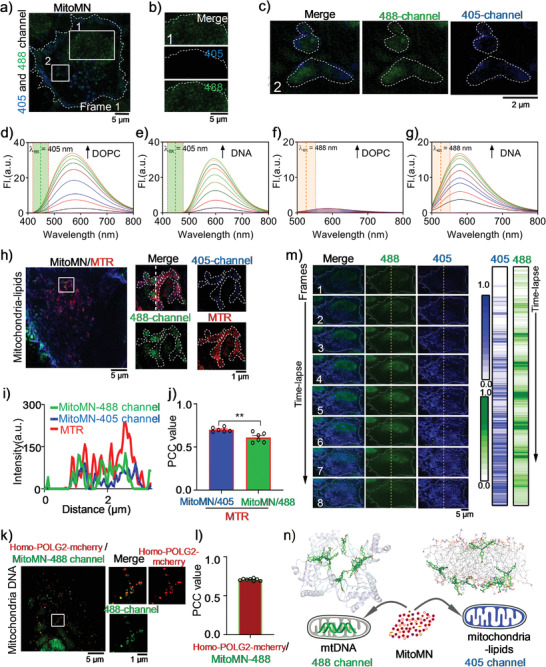
SIM characterizes of MitoMN in living cells. a) HeLa cells stained with **MitoMN** under 405 nm and 488 nm SIM lasers. The dotted line indicates the outline of the cell. b) Zoomed‐in images of white rectangles (labeled 1) in (a). The dotted line indicates the outline of the nucleus. c) Zoomed‐in images of white rectangles (labeled 2) in (a). The dotted line indicates the outline of the mitochondria. d,e) The fluorescence emission spectra of **MitoMN** (10.0 × 10^–6^ m) upon DOPC (180 µg mL^−1^) (d) or DNA (45 µg mL^−1^) (e) titration in PBS (phosphate buffered saline, pH = 7.4), under 405 nm excitation. f,g) The fluorescence emission spectra of **MitoMN** (10.0 × 10^–6^ m) upon DOPC (180 µg mL^−1^) (f) or DNA (45 µg mL^−1^) (g) titration in PBS (pH = 7.4), under 488 nm excitation. h) Mitochondria co‐stained with Mito‐Tracker Red (MTR) and **MitoMN**. The dotted line indicates the outline of the mitochondria. i) Fluorescence intensity profiles in dotted white lines from (h). j) Pearson correlation coefficient (PCC) value for MTR and **MitoMN**. k) Mitochondria co‐stained with **MitoMN** and homo‐POLG2‐mcherry. l) The PCC value for **MitoMN** and homo‐POLG2‐mcherry. m) Photobleaching of **MitoMN** with the continuous irradiation by 405 nm and 488 nm lasers. **Left**: Images of HeLa cells stained with **MitoMN** under continuous illumination at 405 nm and 488 nm, respectively. **Right**: The normalized fluorescence intensity changes of **MitoMN** under 405 nm and 488 nm irradiation. The data were collected from the yellow dotted lines in the left zoom. The raw SIM images were collected with fast 272 MHz for 405 nm and 488 nm SIM lasers, and SIM frames were spaced at 24 s intervals, and the first frame was captured at the 24^th^ second. For SIM imaging, the output powers at the fiber end: 65 mW. n) Schematic representation of **MitoMN** staining mtDNA and mitochondrial lipids under 488 nm SIM laser or 405 nm SIM laser, respectively. The levels of significance were set at n.s. (no significant difference), *p < 0.05, **p < 0.01, ***p < 0.001, and ****p < 0.0001. Data are presented as mean ± SEM (*n* = 6 images for j, and *n* = 8 images for l).

**MitoMN** presented negligible cytotoxicity to living A549 and HeLa cells lines in the concentration range of 0–100 µM for 24 h (Figure S17, Supporting Information). To characterize the imaging characteristics of **MitoMN** in living cells, HeLa cells were stained with **MitoMN** for 6 h, after which they were directly observed under structured illumination microscopy (SIM).^[^ ^21^ ^]^ Results showed that **MitoMN** could be enriched in the periphery of the cell membrane (**Figure** **1**a) and clearly image other components of the matrix (Figure 1a, labeled 1 and 2) under 405‐nm and 488‐nm SIM lasers. Meanwhile, a solid circle (Figure 1a, labeled 1) could be imaged only under a 488‐nm SIM laser (Figure 1b), whereas rod‐shaped particles (Figure 1a, labeled 2) could be imaged with different fluorescence intensities (Figure 1c) under 405‐nm or 488‐nm SIM lasers. The images of HeLa cells using identical imaging conditions in the absence of **MitoMN** indicated the autofluorescence would not interfere with the **MitoMN** signal in living cells (Figure S18, Supporting Information). To confirm which organelles were labeled shown in Figure 1a (labeled 1 and labeled 2), spectroscopic experiments were first performed using phospholipids and nucleic acids, represented by 1, 2‐dioleoyl‐sn‐glycero‐3‐phosphocholine (DOPC), and calf thymus DNA (ctDNA) respectively. **MitoMN** has weak autofluorescence (fluorescence quantum yield (*ɸ*) = 0.007) in buffer solution, while after adding those two biological macromolecules, a significant fluorescence enhancement was detected under 405‐nm excitation (*ɸ* = 0.249 for **MitoMN** + DOPC, and *ɸ* = 0.254 for **MitoMN** + ctDNA) (Figure 1d,e, and Table S1, Supporting Information). The strong combination of **MitoMN** and DOPC/DNA is accompanied by significant fluorescence emission, providing a solid foundation for the continuous addition strategy. Beyond that, the fluorescence obtained from the **MitoMN**–DOPC solution had a broader emission band and demonstrated higher fluorescence intensity at 400–500 nm (Figure S19, Supporting Information), which provided a fluorescence spectral window for selecting lipids from nucleic acids. Similar results were verified in other DNA, including mtDNA and G4‐DNA (Figure S20, Supporting Information), while **MitoMN** did not respond to protein (Figure S21, Supporting Information). Furthermore, under the longer excitation at 488 nm, **MitoMN** showed weaker fluorescence after DOPC was added. By contrast, its fluorescence improved significantly after interacting with ctDNA (Figure 1f,g, and Figure S22, Supporting Information), which paralleled the phenomenon detected by the 488‐nm SIM laser channel (Figure 1c). The difference in fluorescence spectra under the different excitations (*λ*_ex_: 405 nm and *λ*_ex_: 488 nm) provides preliminary evidence that the robust dual‐color response of **MitoMN** is from nucleic acids and lipids. As shown in Figure S23 and Tables S2–S5, Supporting Information, the molecular modeling results show that the combination of **MitoMN** and the simulated lipid membrane was disordered and messy, which prompted the formation of broader fluorescence emission. The probe's sensitive response to DNA, however, can be attributed to the **MitoMN** embedded in DNA's minor hydrophobic grooves, and the regular combination caused the complex to form a sharper, narrower fluorescence emission. That binding form of **MitoMN** is reflected not only in mtDNA but also in other nucleic acids, including dsDNA and G4‐DNA.

Further super‐resolution imaging experiments using a commercial nuclear probe, DAPI,^[^ ^22^ ^]^ suggest that **MitoMN** could image the nuclear matrix in labeled 1 (Figure S24, Supporting Information) under the 488‐nm SIM laser. Under 405‐nm and 488‐nm SIM lasers, the rod‐like structure shown in Figure 1h exhibited high overlap (Figure 1i) and co‐localization (Figure 1j) with a commercial mitochondrial lipid probe, Mito‐Tracker Red (MTR). Added to that, MTR‐colocalized, **MitoMN**‐labeled blue fluorescence intensity exceeded the **MitoMN**‐labeled green fluorescence intensity (Figure 1h), which indicated that the **MitoMN**‐labeled green fluorescence was located in other parts of mitochondrial matrices. Further experiments addressing co‐localization using a homo‐POLG2‐mcherry plasmid^[^ ^23^ ^]^ confirmed that the **MitoMN**‐labeled green fluorescence came from mtDNA with a high co‐localization value (Figure 1k,l). Similar to the difference shown by the spectrum of fluorescence emission, when the emission wavelength exceeded 500 nm, only green fluorescence from mtDNA was detectable; however, when the emission wavelength fell below 500 nm, only blue fluorescence appeared in mitochondrial lipids (Figure S25, Supporting Information). Those results suggest that **MitoMN** can simultaneously illuminate mitochondrial lipids (i.e., in blue with a 405‐nm SIM laser at emission < 500 nm) and mtDNA (i.e., in green with a 488‐nm SIM laser at emission > 500 nm), which provides convenient conditions for the ultra‐precise exploration of mitochondrial events caused by oncosis.

To verify the probe's resistance to photobleaching, **MitoMN‐**stained HeLa cells were investigated under continuous 405‐nm and 488‐nm SIM lasers (Figure 1m). With the extension of irradiation time, the fluorescence intensity from the green channel increased, then gradually decreased; whereas the blue fluorescence intensity uncharacteristically increased throughout the imaging process. Its multi‐positively charged, amphiphilic structure ensured that **MitoMN** can be continuously added into the lipid membranes and thus could resist photobleaching, which may resolve the current imaging bottleneck in the long‐term, dynamic investigations into the biology of mitochondrial lipids. Altogether, with the help of the difference in fluorescence emissions caused by different binding modes between **MitoMN** with lipids and **MitoMN** with DNA, the dual‐color imaging window reasonably accommodated super‐resolution nanoscopy. Using that window, **MitoMN** achieved the dual‐color response imaging of mitochondrial lipids and mtDNA (Figure 1n), as well as labeled mitochondrial lipids in blue with a high degree of photobleaching resistance.

### 2.2 Light Irritation Induces Changing of MitoMN's Fluorescence and Swelling of Mitochondrial

**Figure 2:**
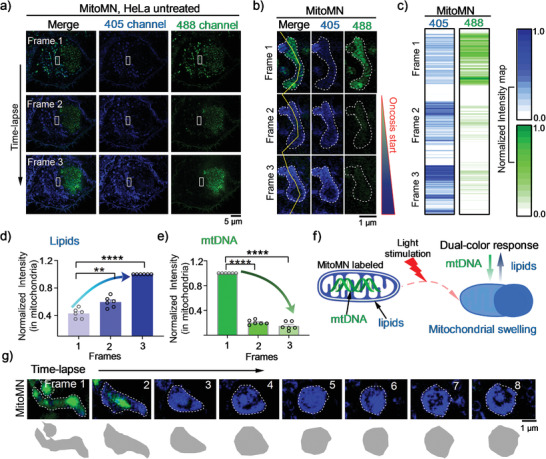
Laser irritation induces changing of MitoMN color and swelling of mitochondria. a) Time‐lapse images of cells stained with **MitoMN** under 405 nm and 488 nm SIM lasers. b) Zoomed‐in images of white rectangles in (a), and yellow dotted lines indicate the region of interest for fluorescence measurement. c) The blue and green fluorescence changes of **MitoMN** under 405 nm and 488 nm SIM lasers. The data were collected from the yellow dotted lines in figure (b). The raw SIM images were collected with fast 272 MHz for 405 nm and 488 nm SIM lasers, and SIM frames were spaced at 24 s intervals, and the first frame was captured at the 24^th^ s. d) Normalized blue fluorescence intensity changes in **MitoMN** stained mitochondria. e) Normalized green fluorescence intensity changes in **MitoMN** stained mitochondria. f) Schematic representation of the dual‐color response of a continuous add‐on probe, **MitoMN**, imaging of mitochondria under 405 nm and 488 nm SIM lasers. g) Time‐lapse images of cells stained with **MitoMN** for tracking mitochondrial swelling and mtDNA damage under 405 nm and 488 nm SIM lasers. The raw SIM images were collected with fast 272 MHz for 405 nm and 488 nm SIM lasers. SIM frames were spaced at 24 s intervals, and the first frame was captured at the 24th second. For SIM imaging, the output powers at the fiber end: 65 mW. The levels of significance were set at n. s. (no significant difference), *p < 0.05, **p < 0.01, ***p < 0.001, and ****p < 0.0001. Data are presented as mean ± SEM (n = 6 mitochondria form 6 cells for (d) and (e)).

After verifying the photophysical properties and target position of **MitoMN** in the mitochondria, the long‐term dynamics of mitochondrial lipids and mtDNA were tracked simultaneously (**Figure** **2**a). After exposure of HeLa cells to 405/488 channels, **MitoMN**‐stained mitochondrial matrix presented strong green fluorescence from mtDNA and blue fluorescence from mitochondrial lipids (Figure S26, Supporting Information). With the extension of light stimulation, the **MitoMN**‐stained mitochondrial matrix displayed a dual‐color response (Figure 2b), in which the green fluorescence intensity from mtDNA gradually decreased, while the blue fluorescence intensity from lipids gradually increased (Figure 2c–e). In addition, when the green fluorescence was quenched, mitochondria began to show morphological changes in the form of swelling (Figure 2b–g), thereby suggesting that formerly green mtDNA had been lost, and that **MitoMN** had initiated the process of mitochondrial destruction.

To clarify the association of **MitoMN** to mtDNA under light irradiation, the fluorescence lifetime spectroscopy measurements of **MitoMN** with different forms of DNA in the dark or under 425‐nm LED irradiation were performed. As shown in Figures S27 and S28, Supporting Information, compared to the fluorescence lifetime under dark conditions, an extended fluorescence lifetime was observed after irradiation in the **MitoMN**–DNA solution, which implies an enhanced mtDNA binding status with **MitoMN** under light irradiation.^[^ ^24^ ^]^ Further DNA photocleavage experiments showed that upon incubation of pBR322 plasmid DNA with **MitoMN** under dark condition, a hindrance of gel mobility of supercoiled DNA was observed. Upon light irradiation, **MitoMN** induced the significant cleavage of the supercoiled conformation, and the intensity of the nicked band rose, thereby indicating that **MitoMN** has potent activity in DNA cleavage under irradiation (Figure S29, Supporting Information). In further experiments, the presence of 8‐oxoguanine (8‐oxo‐dG) was detected by immunofluorescence in HeLa cell lines (Figure S30, Supporting Information). Compared with a series of control experiments, in which HeLa cells were incubated with DMSO under dark or irradiative conditions, the HeLa cells incubated with **MitoMN** under dark conditions did not produce the elevated expression of 8‐oxo‐dG, whereas cells incubated with **MitoMN** under light conditions showed a significant elevation in 8‐oxo‐dG expression in both mitochondria and nuclei. Those results indicate **MitoMN** caused severe damage to the DNA in mitochondria and nuclei under irradiation. With the continuous irradiation of the SIM laser, **MitoMN** induced mtDNA damages accompanied by the green fluorescence from mtDNA quenching—and due to the increase of blue fluorescence caused by the continuous add‐on of **MitoMN** in mitochondrial lipids—led to the emergence of the dual‐color response in the mitochondria (Figure 2f,g). In addition, when green fluorescence disappeared, the marked, enlargement of mitochondria swelling was activated (Figure 2g). It is generally accepted that cells, which suffered with oncosis induction, present with typical events such as membrane blebs, nucleus swelling, chromatin clumping and so on^[^ ^4^, ^5^, ^6^, ^7^ ^]^ (Figure S31a, Supporting Information). These events occurred in the cells treated with **MitoMN**, which are similar to that of NaN_3_‐induced oncosis^[^ ^7e^ ^]^ (Figures S31b and S31c, Supporting Information). The HeLa cells incubated with **MitoMN** under irradiation collected different frames (Figure S31d, Supporting Information) or different random fields (Figure S31e, Supporting Information) also presented similar features. The video collected from **MitoMN** stimulated HeLa cells showed that membrane blebbing can occur within 10 min, which showed that the cell membrane swelling process is induced by oncosis (Videos S1 and S2, Supporting Information). In addition, the annexin V‐FITC and propidium Iodide (PI) double‐staining using the flow cytometry analysis confirmed that the cell membrane swelling process is not induced by apoptosis and necrosis (Figure S32, Supporting Information). The oncosis process was further verified in other cell lines, including two normal cells (Human umbilical vein endothelial cells (HUVEC) and chondrocytes from rabbit (CHS)), and one cancer cell (A549) along with NaN_3_‐assisted oncosis as control through SIM (Figure S33, Supporting Information), which showed similar results after **MitoMN** or NaN_3_ treatment.

### 2.3 Light Activates MitoMN‐Induced Oncosis

It is generally accepted that ROS plays an essential role in mitochondrial damage and participates in numerous biological processes,^[^ ^25^ ^]^ such as causing an acceleration of apoptosis or initiation of autophagy.^[^ ^26^, ^27^ ^]^ To reduce the destruction of cell biological processes by endogenous ROS, some ROS eliminator drugs have been developed, such as the commercial ROS inhibitor Apocynin,^[^ ^28^ ^]^ which targets NADPH oxidase to inhibit the production of ROS, thereby protecting cells.

**Figure 3:**
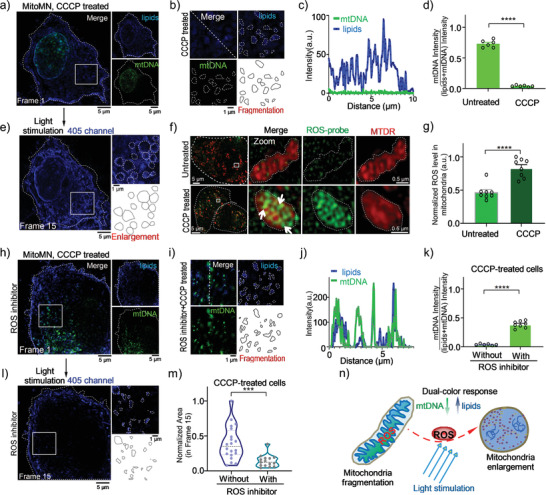
ROS are required for MitoMN induced oncosis. a) CCCP‐treated cells stained with **MitoMN** under 405 nm and 488 nm SIM lasers. b) Zoomed‐in images of white rectangles in (a), white dotted lines indicate region of interest for fluorescence measurement shown in (c), and white circle lines indicate fragmented mitochondria region damaged by CCCP. d) Fluorescence intensity ratio of mtDNA in **MitoMN** stained mitochondria matrix with or without CCCP‐treated. e) Frame 15^th^ image of cells stained **MitoMN** under continuous irradiation by 405 nm SIM laser, white circle lines indicate damage of mitochondria. f) Cells stained with ROS probe DCFH‐DA, and MTDR with or without CCCP‐treated. White arrows represent the particles induced by ROS. g) Normalized ROS level in mitochondria with or without CCCP‐treated. h) Cells treated with ROS inhibitor and CCCP sequentially, and then stained with **MitoMN** under 405 nm and 488 nm SIM lasers. i) Zoomed‐in images of white rectangles in (h), white dotted lines indicate region of interest for fluorescence measurement shown in (j), and white circle lines indicate fragmented mitochondria region, (k) fluorescence intensity ratio of mtDNA in **MitoMN** stained mitochondria matrix with or without ROS inhibitor per‐treatment, CCCP‐treated cells. l) Frame 15^th^ image of cells stained **MitoMN** under continuous irradiation by 405 nm SIM laser, white circle lines indicate enlargement mitochondria. m) Normalized mitochondria area with or without ROS inhibitor pre‐treatment, CCCP‐treated cells. n) Schematic representation of ROS for mitochondria enlargement in oncosis cells. The levels of significance were set at n.s. (no significant difference), *p < 0.05, **p < 0.01, ***p < 0.001, and ****p < 0.0001. Data are presented as mean ± SEM; *n* = 6 images for (d), *n* = 6 to 8 images for (g) and (k), *n* = 20 cells for without ROS inhibitor group in (m), and *n* = 15 cells for with ROS inhibitor group in (m).

To understand whether ROS was involved in **MitoMN**‐induced oncosis, a commercial drug to induce mitochondrial damage, carbonyl cyanide *m*‐chlorophenyl hydrazone (CCCP),^[^ ^29^ ^]^ was used to pretreat cells for 12 h and thus upregulate ROS in mitochondria in the first SIM frame image. Next, the CCCP‐treated cells were stained with **MitoMN**, after which fragmented mitochondria appeared (**Figures** **3**a,b), as consistent with reports of earlier work.^[^ ^17^ ^]^ Similar to the **MitoMN** stained mitochondria in Figure 2g, with the extension of light stimulation, the green mtDNA fluorescence intensity was lower than the blue lipid fluorescence intensity, gradually (Figure S26, Supporting Information and Figure 3c). Compared with untreated cells, the percentage of green fluorescence intensity in the CCCP‐treated cells had decreased significantly (Figure 3d). More importantly, enlarged mitochondria can occur in the CCCP‐treated cells under continuous 405‐nm SIM laser excitation (Figure 3e, enlargement). To further verify CCCP's effect on the level of ROS in mitochondria, Mito Tracker Deep Red (MTDR),^[^ ^30^ ^]^ and a commercial ROS probe, DCFH‐DA,^[^ ^31^ ^]^ were used to co‐stain cells (Figure 3f). SIM imaging showed that the particles induced by ROS located in the mitochondrial cristae in CCCP‐treated cells (Figure 3f enlargement), and that the level of ROS throughout mitochondria was higher than that in untreated cells (Figure 3g). Therefore, mitochondria cristae were damaged under high ROS level. Meanwhile, the analysis of cells with or without CCCP treatment (Figure S34, Supporting Information) shows that the CCCP can accelerate MitoMN‐induced mitochondrial enlargement.

We performed further experiments with the ROS generation inhibitor, Apocynin,^[^ ^28^ ^]^ to pretreat cells for 12 h before another 12 h CCCP treatment, and then to treat with **MitoMN** (Figure 3h,i). Results indicated that the green fluorescence from mtDNA was stable present in mitochondria (Figure 3j) and that the percentage of green fluorescence intensity was significantly increased in the cells pretreated with ROS inhibitor (Figure 3k). On top of that, when the production of endogenous ROS in mitochondria was inhibited, the efficiency of **MitoMN**‐induced mitochondrial enlargement was inhibited as well (Figure 3l), and the area demonstrating oncosis of cells was blocked (Figure 3m). Those results confirm that the additional ROS can accelerate **MitoMN**‐induced mitochondrial enlargement, and can efficiently induce oncosis (Figure 3n). Due to the suitable ROS generation ability of **MitoMN** shown in Figure S16, Supporting Information, the results also demonstrate the potential of using **MitoMN** to damage cancer cells through light‐activated oncosis.

### 2.4 MitoMN Reveals a Nonlinear Enlargement of Mitochondria in Oncosis

**Figure 4:**
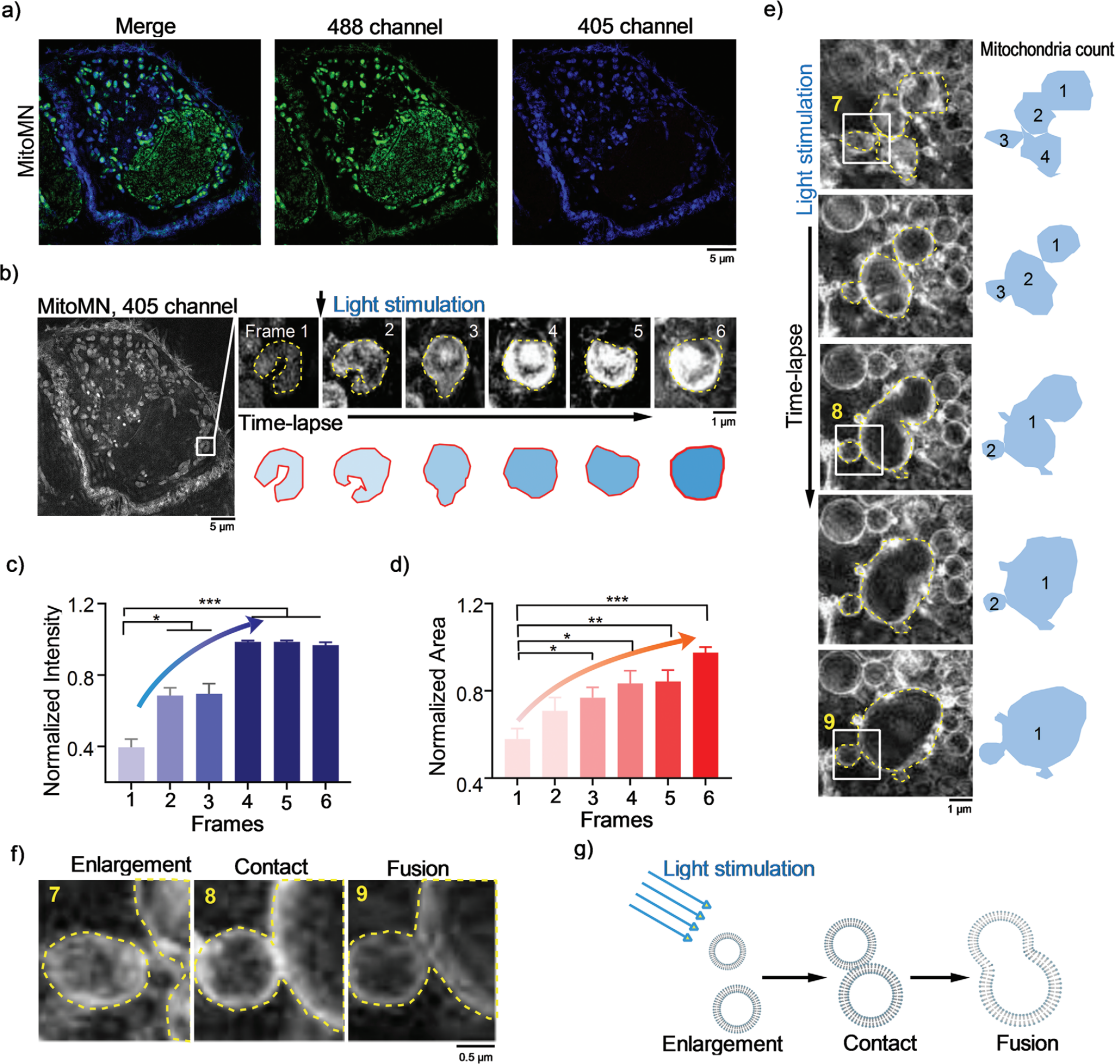
MitoMN reveals a nonlinear mitochondria enlargement in oncosis. a) Cells stained with **MitoMN** under 405 nm and 488 nm SIM lasers. b) SIM tracking of mitochondria enlargement in oncosis under continuous irradiation by 405 nm SIM laser. c,d) Normalized fluorescence intensity (c) and area (d) collected from the **MitoMN** stained mitochondria. e) **MitoMN** imaged a nonlinear mitochondria enlargement in oncosis; f) Zoom‐in images of white rectangles in (e), green plaque shown several nonlinear mitochondria enlargement in oncosis under continuous irradiation by 405 nm SIM laser. g) Schematic representation of continuous irradiation induces a nonlinear mitochondria enlargement by **MitoMN**. The raw SIM images were collected with fast 272 MHz for 405 nm laser, SIM frames were spaced at 24 s intervals, and the first frame was captured at the 24^th^ second. The dotted line indicates the outline of the mitochondria. For SIM imaging, the output powers at the fiber end: 65 mW. The levels of significance were set at n.s. (no significant difference), *p < 0.05, **p < 0.01, ***p < 0.001, and ****p < 0.0001. Data are presented as mean ± SEM (*n* = 3 mitochondria from 3 cells in (c), and *n* = 4 mitochondria from 4 cells in (d)).

Last, long‐term imaging of individual mitochondria stained with **MitoMN** was performed to detect the oncosis duration, and clarify whether interaction occurred between individual mitochondria during oncosis (**Figures** **4**a,b). Under the continuous excitation of a 405‐nm SIM laser, individual mitochondria showed a morphological change from being fibrous‐like to being enlarged, accompanied by an increase in fluorescence intensity (Figure 4c) and an expanded mitochondrial area (Figure 4d), and **MitoMN**‐induced mitochondrial swelling events could not be activated by other inducers (Figure S35, Supporting Information). In addition, when mitochondria showed single instances of enlargement, multiple enlarged mitochondria could interact with each other under light stimulation, which was characterized by the fusion of four independent mitochondria into one, large mitochondria for further nonlinear swelling (Figure 4e). SIM clearly recorded the occurrence of fusion events of mitochondria, which would be a unique indicator of irreversible oncosis (Figure 4f).

The whole process of light‐activated oncosis induced by **MitoMN** could be described in three stages: before oncosis (i.e., when **MitoMN** stained mitochondria with blue and green fluorescence, when the green fluorescence loss indicates the beginning of oncosis, and when mitochondria begin to accumulate ROS), initial oncosis (i.e., when **MitoMN** is under light stimulation to produce high level of ROS that damages mitochondria's structure to accelerate individual fibrous‐like mitochondria to enlarged mitochondria), and accelerated oncosis (i.e., when multiple mitochondria interact with each other to fuse, then form larger mitochondria to promote cell death). During initial oncosis, the expansion of mitochondria is linear and reversible, while the fusion of enlarged mitochondria further pushes the swelling into a nonlinear regime for inducing irreversible oncosis.

## 3 Conclusion

In summary, a continuous add‐on probe, **MitoMN**, was designed to resolve the bottleneck in examining the dynamic process of oncosis that was previously unobservable with current mitochondrial probes. Characterized by enhanced fluorescence under light stimulation, **MitoMN** allows capturing the entire process of mitochondrial dynamics in oncosis and can image mitochondria with a dual‐color response, such that when the green disappeared, the mitochondria commenced enlargement. More importantly, the probe has revealed a novel event for the first time: how enlarged mitochondria interact and fuse with each other to irreversibly accelerate overall oncosis. For those reasons, this tool seems exceptionally useful for staging oncosis (i.e., before oncosis, initial oncosis, and accelerated oncosis), which may result in a range of future new studies on the biological details of oncosis. Finally, since the **MitoMN** has the potential to induce the death of cancer cells through light‐activated oncosis, our study also demonstrates that subcellular dynamics could be used to quantify oncosis at the sub‐cellular level in photodynamic diagnosis and treatment.

## 4 Experimental Section

### Cell Culture

HeLa cells were gifted from Dr. Chunyin Liu's lab (Shandong First Medical University, Shandong Province, P. R. China); Human umbilical vein endothelial cells (HUVEC), chondrocytes from rabbit (CHS), and A549 cells were obtained from Dr. Peixue Ling's lab (Shandong University, Shandong province, PR. China). HeLa, A549, HUVEC, and CHS cells were cultured in DMEM supplemented with 10% fetal bovine serum (FBS, VivaCell, Shanghai, China), penicillin (100 units per mL), and streptomycin (10 000 units per mL) in a 5% CO_2_ humidified incubator at 37 °C.

### Cell Transfection

2400 ng of DNA were combined with 7.2 µL of Turbofect transfection solution (Thermo Fisher Scientific) in 240 µL of serum‐free DMEM medium. The transfection mixtures were incubated at room temperature for 20 min prior to adding to cells cultured in 35 mm dishes with 500 µL complete medium. After 3 h of transfection, the transfection medium was replaced with 1 mL complete medium supplemented with penicillin–streptomycin solution. And 16 h after transfection of living HeLa cells further stained with **MitoMN** for co‐localization assay.

### Cell Treatment and Staining

For **MitoMN** labeling assay, cells were seeded on a glass‐bottom micro‐well dish and incubated with 2 mL of DMEM supplemented with 10% FBS for 24 h, and then stained with **MitoMN** (10.0 × 10^–6^ m) in the phenol‐free medium for 3 h. For ROS inhibitor assay, the cells were pre‐treated with 20.0 × 10^–6^ m apocynin for 12 h, and then further treated with 10.0 × 10^–6^ m CCCP for 12 h; after treatment, cells were stained with **MitoMN** (10.0 × 10^–6^ m) in phenol‐free medium for 3 h; for co‐localization assay, cells were stained with DAPI or MTR (0.1 × 10^–6^ m), and then stained with **MitoMN** (10.0 µm) in phenol‐free medium for 3 h; for ROS detecting assay, the cells were stained with 10.0 × 10^–6^ m ROS probe, DCFH‐DA, for 30 min and further stained with MTDR (0.1 × 10^–6^ m) for 30 min, then cells were treated with or without 10.0 × 10^–6^ m CCCP for 12 h; for mtDNA co‐localization assay, cells were stained with homo‐POLG2‐mcherry plasmid for 16 h, and then stained with **MitoMN** (10.0 × 10^–6^ m) in the phenol‐free medium for 3 h; for oncosis, cells were treated with 1% NaN_3_ for 3 h; for apoptosis, cells were treated with 0.5 × 10^–3^ m H_2_O_2_ for 24 h; and for ferroptosis, cells were treated with 20.0 × 10^–6^ m erastin for 12 h, and further stained with MTDR (0.1 × 10^–6^ m) for 30 min. Finally, cells were observed under an OMX‐3D‐SIM super‐resolution microscope (GE, USA).

### Super‐Resolution Microscopy Imaging

Super‐resolution images were acquired on a commercial OMX‐3D‐SIM Microscope. Images were obtained at 512 × 512 using Z‐stacks with a step size of 0.125 µm. Raw SIM image collection parameters: SIM 405 channel: *λ*_ex_ = 405 nm, *λ*(max)_em_ = 447 nm (417–476 nm). The model was set to fast 272 MHz, the gain was set as 1, the exposure was set to 150, and the percentage of transmission was set to 7.0%. SIM 488 channel: *λ*_ex_ = 488 nm, *λ*(max)_em_ = 525 nm (500–550 nm). The laser model was set to fast 272 MHz, the gain was set to 1, the exposure was set to 20, and the percentage of transmission was set as 7.0%, SIM frames were spaced at 24 s intervals for time‐lapse assay. For SIM imaging, the output powers at the fiber end: 65 mW. All fluorescence images were analyzed, and their backgrounds were subtracted with ImageJ software.

### Photobleaching Assay

Photobleaching experiments were performed on a commercial OMX‐3D‐SIM Microscope with a 100× oil immersion objective. The cells were stained with **MitoMN** and then exposed to OMX‐3D‐SIM lasers intensity of 100% 405 nm and 488 nm. The image was obtained at a 30 s interval. SIM images were analyzed with ImageJ software.

### Statistical Analysis

Statistical analysis was performed with Prism 8 (GraphPad). All biological experiments were performed at least twice with triplicates in each experiment. Normality and lognormality test was conducted. In the case of normal distribution, the statistical comparison of results was checked with a Student's *t* test. In the case of non‐normal distribution, the statistical comparison of results was checked with a Mann–Whitney test. The levels of significance were set at n. s. (no significant difference), *p < 0.05, **p < 0.01, ***p < 0.001, and ****p < 0.0001. Representative results were depicted in this report, and data were presented as means ± SEM with statistical significance. Statistical significances and sample sizes in all graphs are indicated in the corresponding figure legends.

## Conflict of Interest

The authors declare no conflict of interest.

## Supporting information

Supporting Information

Supplemental Video 1

Supplemental Video 2

## Data Availability

The data that support the findings of our study are available from the corresponding author upon reasonable request.
