# Supplementary material for: A Continuous Add‐On Probe Reveals the Nonlinear Enlargement of Mitochondria in Light‐Activated Oncosis
Source: Adv Sci (Weinh). 2021 Jul 1;8(17):2004566. doi: 10.1002/advs.202004566 (PMC8425930; doi:10.1002/advs.202004566)
Supplement: Supplementary file 1 — Supporting Information [file ADVS-8-2004566-s003.pdf]

© 2021 Wiley-VCH GmbH

ADVANCED SCIENCE
Open Access

Supporting Information

for *Adv. Sci.*, DOI: 10.1002/advs.202004566

A continuous add-on probe reveals the nonlinear enlargement of mitochondria in light-activated oncosis

Kang-Nan Wang , Xintian Shao, Zhiqi Tian, Liu-Yi Liu, Chengying Zhang, Cai-Ping Tan, Jie Zhang, Peixue Ling, Fei Liu, Qixin Chen, Jiajie Diao*, Zong-Wan Mao**

Supporting Information
©Wiley-VCH 2019
69451 Weinheim, Germany

A continuous add-on probe reveals the nonlinear enlargement of mitochondria in light-activated oncosis

Kang-Nan Wang[#] , Xintian Shao[#], Zhiqi Tian[#], Liu-Yi Liu[#], Chengying Zhang[#], Cai-Ping Tan, Jie Zhang, Peixue Ling, Fei Liu, Qixin Chen*, Jiajie Diao*, Zong-Wan Mao*

Table of Contents

Experimental Procedures 4
Figure S1. ^{1}H NMR spectrum of S1........ 10
Figure S2. ^{13}C NMR spectrum of S1........ 10
Figure S3. ^{1}H NMR spectrum of S2........ 11
Figure S4. ^{13}H NMR spectrum of S2. 11
Figure S5. ^{1}H NMR spectrum of S3........ 12
Figure S6. ^{13}C NMR spectrum of S3........ 12
Figure S7. ^{1}H NMR spectrum of S5**.**........ 13
Figure S8. ^{13}C NMR spectrum of S5........ 13
Figure S9. ^{1}H NMR spectrum of MitoMN**.**........ 14
Figure S10. ^{13}C NMR spectrum of MitoMN**.**........ 14
Figure S11. EMS spectrum of MitoMN**.**........ 15
Figure S12. UV-Vis absorption and fluorescence emission spectra of MitoMN........ 15
Figure S13. Molecular orbital amplitude plots of the HOMO and LUMO energy levels of MitoMN. 16
Figure S14. The selectivity of MitoMN for reactive oxygen species........ 16
Figure S15. The selectivity of MitoMN for bioactive anion/cations........17
Figure S16. Rate of decay of ABDA sensitized by control and MitoMN in PBS 17
Figure S17. Viability of HeLa/A549 cells treated with the various concentrations of MitoMN. 18
Figure S18. HeLa cells stained without or with MitoMN under 405 and 488 SIM lasers........ 18
Figure S19. The emission fluorescence spectra of MitoMN upon DOPC or ctDNA under 405 nm excitation..... 19
Figure S20. The emission fluorescence spectra of MitoMN upon DOPC or G4-DNA under 405 nm excitation . 19
Figure S21. The emission fluorescence spectra of MitoMN upon protein under 405 nm excitation........ 20
Figure S22. The UV-vis spectra and emission spectra of MitoMN upon DOPC or dsDNA under 488 nm excitation. 20
Figure S23. The molecular modeling calculations based on the optimized structure of MitoMN and DOPC/DNA. 21
Figure S24. Nucleus stained with DAPI and MitoMN. 22
Figure S25. MitoMN labeled mitochondria matrix under different SIM emission and excitation. 22
Figure S26. The fluorescence distribution of MitoMN stained lipids and mtDNA in HeLa untreated cells. 23
Figure S27. Fluorescence lifetime spectra changes of MitoMN binding with biomacromolecules under dark conditions. 24
Figure S28. Fluorescence lifetime changes of MitoMN binding with biomacromolecules under dank or light conditions 24
Figure S29. Electrophoresis experiments of DNA photo-cleavage of pBR322 plasmid DNA treated with MitoMN under dark or irradiation conditions 25
Figure S30. ROS produced by MitoMN under irradiation is associated with the accumulation of 8-oxodG........ 25
Figure S31. Comparison of nuclei and membrane morphology under MitoMN and NaN3 stimulation. 26

Figure S32. Annexin V-FITC/PI double staining analyzed by flow cytometry. 26

Figure S33. Different living cell lines treated with NaN_3 or MitoMN 26

Figure S34. The with or without CCCP-treated cells stained with MitoMN under light stimulation.................... 29

Figure S35. The untreated cells, NaN_3 induced oncosis cells and the Erastin treated cells had noticeable changes in mitochondrial morphology.. 30

Table S1. Photophysical properties of MitoMN at room temperature. 31

Table S2. The representative docked free energies of the docking models between MitoMN and simulated membrane.. 31

Table S3. The representative docked free energies of the docking models between MitoMN and mtDNA.......... 32

Table S4. The representative docked free energies of the docking models between MitoMN and dsDNA. 32

Table S5. The representative docked free energies of the docking models between MitoMN and G4-DNA........ 33

References.. 34

Experimental Procedures

Materials and instruments

All reagents and solvents (analytical grade) were used as received from commercial sources unless otherwise indicated. Carbonyl cyanide 3-chlorophenylhydrazone (CCCP) was obtained from Thermo Fisherscientific (Grand Island, NY, USA); Apocynin was obtained from Med Chem Express (NJ, USA); Mito Tracker Red CMXRos (MTR) were obtained from Thermo Fisherscientific (Grand Island, NY, USA). Nuclear probe (DAPI) and ROS probe (DCFH-DA) were obtained from Beyotime Biotechnology (Shanghai, PR China); Penicillin-streptomycin (10,000 units/ml), Fetal bovine serum (FBS), Dulbecco's modified Eagle's medium (DMEM), phenol-free DMEM and other cell culture reagents were obtained from Gibco BRL (Grand Island, NY, USA); 3-(4,5-Dimethylthiazol-2-yl)-2,5-diphenyltetrazolium bromide (MTT) was obtained from Sigma Aldrich; DMSO, Hoechst 33342 (2'-(4- ethoxyphenyl)-5-(4-methyl-1-piperazinyl)-1H,3'H-2,5'-bibenzimidazole, were obtained from Sigma Aldrich; Rose Bengal (RB), Rhodamine 6G, DOPC (1, 2-dioleoyl-sn-glycero-3-phosphocholine), bromoethane, and protein (BSA, bovine serum albumin) were obtained from Energy Chemical; PI (propidium iodide), Erastin was obtained from MedChemExpress (NJ,USA), NaN_3 were obtained from Sigma Aldrich and used as received. The tested compounds were dissolved in DMSO before the experiments, and the concentration of DMSO was 1% (v/v). Homo-POLG2-mcherry plasmid was purposed from Weizhen Biosciences Ins (Shandong, PR China). ^{1}H NMR and ^{13}C NMR spectra were recorded on a Mercury Plus 400 or 500 spectrometers. Shifts are referenced relative to the internal solvent signals. ESI-MS were recorded on a Thermo Finnigan LCQ DECA XP spectrometer. The quoted m/z values represent the major peaks in the isotopic distribution. UV–vis spectra were recorded on a Varian Cary 300 spectrophotometer (USA). Fluorescence microscopy of cells was performed in Carl Zeiss LSM 710. For MTT assays, the absorbance was quantified using the Infinite M200 microplate reader.

Synthesis and Characterization

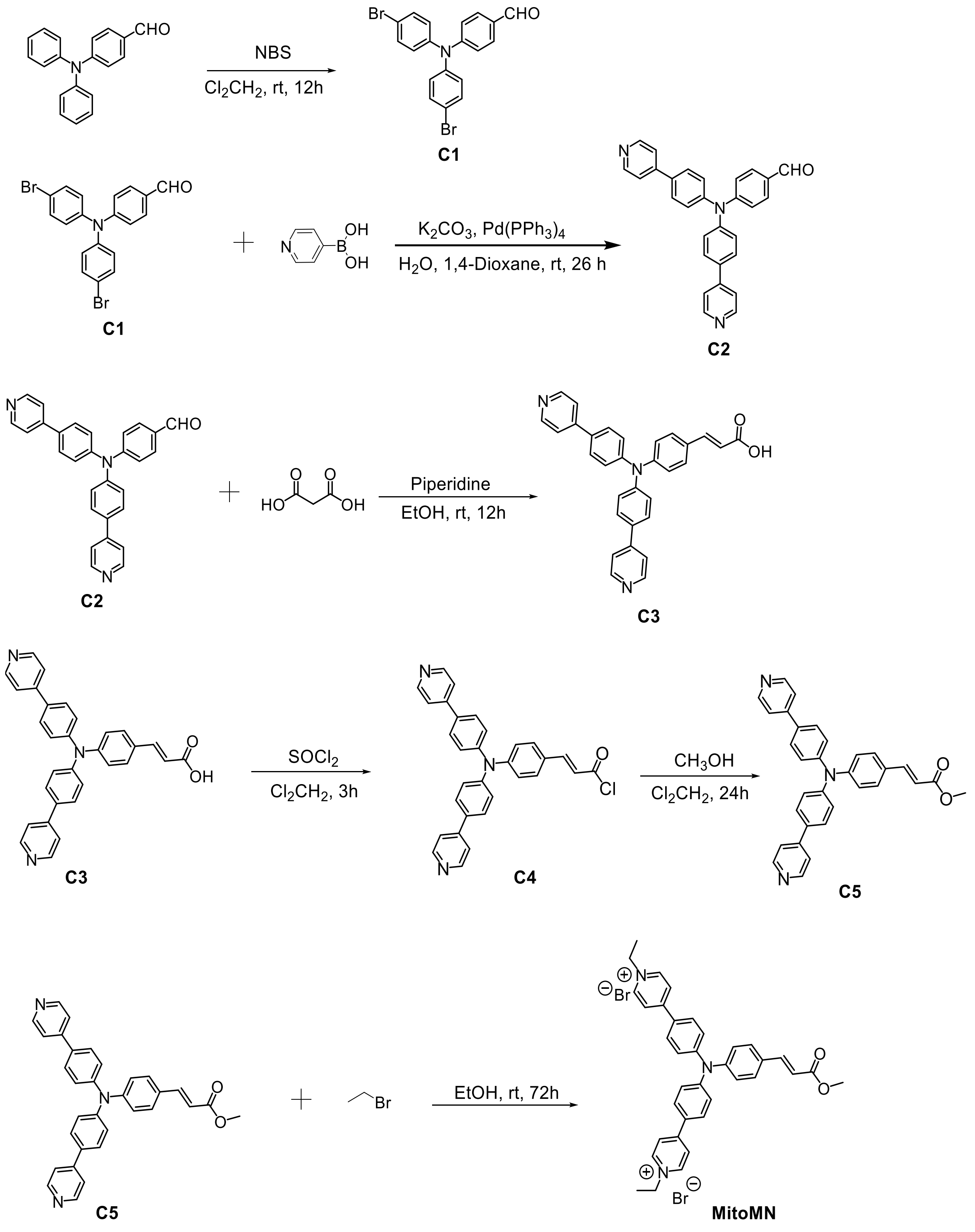

Scheme S1. Synthetic routes to **MitoMN**.

The synthesis of compounds **C1** and **C2** was followed by literature.[1]

C1: light yellow solid, 2.15 g, 69 % yield: ^{1}H NMR (400 MHz, DMSO) δ 9.82 (s, 1H), 7.77 (d, *J* = 8.8 Hz, 2H), 7.57 (d, *J* = 8.8 Hz, 4H), 7.11 (d, *J* = 8.8 Hz, 4H), 7.00 (d, *J* = 8.7 Hz, 2H). ^{13}C NMR (101 MHz, DMSO) δ 191.51 (s), 152.52 (s), 145.47 (s), 133.57 (s), 132.00 (s), 130.38 (s), 128.57 (s), 120.48 (s), 118.10 (s).

C2: light yellow solid, 0.42 g, 74 % yield: ^{1}H NMR (500 MHz, $CDCl_3$) δ 9.88 (s, 1H), 8.67 (d, *J* = 8.6 Hz, 4H), 7.77 (d, *J* = 8.7 Hz, 2H), 7.64 (d, *J* = 8.6 Hz, 4H), 7.52 (d, *J* = 5.5 Hz, 4H), 7.29 (d, *J* = 8.6 Hz, 4H), 7.19 (d, *J* = 8.6 Hz, 2H). ^{13}C NMR (126 MHz, $CDCl_3$) δ 190.48 (s), 152.43(s), 150.29 (s), 147.29 (s), 147.02 (s), 134.36 (s), 131.41 (s), 130.60 (s), 128.38 (s), 126.09 (s), 121.50 (s), 121.22 (s).

(E)-3-(4-(Bis(4-(pyridin-4-yl)phenyl)amino)phenyl)acrylic acid (C3): 0.76 g (1.78 mmol) **C2** and 0.554 g (3 eq.) malonic acid were added to a round bottom flask containing 20 mL of CH_3CN, and 1.23 mL (7 eq. 1.058 g) of piperidine was added subsequently. The reaction mixture was heated at 82 ^{o}C for 12 h. After the reaction was cooled to room temperature, 100 mL of water was added into the reaction solution. The precipitate of compound **C3** was filtered under reduced pressure and washed with ethanol to yield **C3** as light-yellow solid (0.75 g, 92% yield). ^{1}H NMR (400 MHz, DMSO) δ 12.27 (s, 1H), 8.62 (d, *J* = 4.9 Hz, 4H), 7.83 (d, *J* = 7.5 Hz, 4H), 7.66 (d, *J* = 4.7 Hz, 4H), 7.56 (d, *J* = 8.3 Hz, 2H), 7.44 (d, *J* = 15.8 Hz, 1H), 7.22 (d, *J* = 8.6 Hz, 4H), 7.09 (d, *J* = 8.5 Hz, 2H), 6.43 (d, *J* = 15.8 Hz, 1H). ^{13}C NMR (101 MHz, DMSO) δ 150.68 (s), 147.81 (s), 146.61 (s), 132.33 (s), 130.51 (s), 129.47 (s), 128.61 (s), 124.88 (s), 124.52 (s), 121.12 (s), 118.23 (s).

Methyl (E)-3-(4-(bis(4-(pyridin-4-yl)phenyl)amino)phenyl)acrylate (C5): 0.47 g (1 mmol) of **C3**, 10 mL of thionyl chloride and a few drops of pyridine were added to a round bottom flask with condenser and gas absorption device. The mixture was stirred at -5 ^{o} C for 3 h. The reduced hydrochloric acid mist was received by 5% NaOH solution. The precipitate was filtered and CH_3OH (20 mL) was added into a round bottom flask, continue to react for 24 hours. After reaction, $NaHCO_3$ (1 M) was added to remove the unreacted thionyl chloride and hydrochloric acid. The slight yellow precipitate was filtered and repeatedly washed with 100 mL water and 100 mL diethyl ether three times. The residue was purified by column chromatography over silica gel to yield **C5** as light-yellow solid (0.42 g, 87 % yield). ^{1}H NMR (400 MHz, DMSO) δ 8.62 (s, 4H), 7.83 (d, *J* = 7.6 Hz, 4H), 7.71 (s, 4H), 7.64 (d, *J* = 16.4 Hz, 2H), 7.23 (d, *J* = 7.6 Hz, 4H), 7.09 (d, *J* = 7.7 Hz, 2H), 6.54 (d, *J* = 15.9 Hz, 1H), 3.72 (s, 3H). ^{13}C NMR (101 MHz,

DMSO) δ 167.30 (s), 150.69 (s), 148.81 (s), 147.59 (s), 146.57 (s), 144.31 (s), 132.71 (s), 130.43 (s), 129.17 (s), 128.68 (s), 125.28 (s), 123.65 (s), 121.17 (s), 116.49 (s), 51.85 (s).

(E)-4,4'-(((4-(3-methoxy-3-oxoprop-1-en-1-yl)phenyl)azanediyl)bis(4,1-phenylene))bis(1-ethylpyridin-1-ium) (MitoMN): **MitoMN** was obtained as Brick red solid (454 mg, 65% yield) by treatment of **C5** (483 mg) with 20 equivalent bromoethane under 75 ^{o}C in ethanol for 72 h. ESI-MS (m/z): calcd. for $[M-2Br]^{2+}$ ($C_{36}H_{35}N_3O_2^{2+}$): 541.27; found: 541.27 ^{1}H NMR (400 MHz, DMSO) δ 8.86 (s, 4H), 8.22 (d, J = 5.6 Hz, 4H), 8.11 (d, J = 8.7 Hz, 1H), 8.03 (d, J = 8.7 Hz, 4H), 7.78 (t, J = 8.7 Hz, 2H), 7.34 – 7.22 (m, 6H), 6.63 (d, J = 5.5 Hz, 1H), 4.63 – 4.55 (m, 4H), 3.74 (s, 3H), 1.56 (t, J = 7.3 Hz, 6H). ^{13}C NMR (126 MHz, DMSO) δ 167.83 (s), 154.84 (s), 150.02 (s), 147.38 (s), 145.08 (s), 143.80 (s), 131.42 (s), 129.76(s), 129.57(s), 128.29(s), 125.84(s), 124.63(s), 123.63(s), 117.12(s), 58.65(s), 51.14(s), 16.37(s).

Optical properties. Steady-state emission spectra and lifetime measurements were conducted on a combined fluorescence lifetime and steady-state spectrometer FLS 920 (Edinburgh). UV–vis spectra were recorded on a Varian Cary 300 spectrophotometer (USA). The spectra were carried out in buffer solution (10.0 mM Tris-HCl buffer, 100 mM KCl, pH 7.4). Stock solutions of **MitoMN** (10.0 mM) were prepared in DMSO and diluted to the indicated concentrations by PBS. Other analytes (ClO^-, H_2O_2, $ONOO^-$, O_2^-, CH_3COO^-, Cl^-, OH^-, CO_3^{2-}, F^-, I^-, NO^{2-}, PO_3^{2-}, SCN^-, SO_3^{2-}, SO_4^{2-}, Al^{3+}, Ca^{2+}, Na^+ Mn^{2+}, Cu^{2+}, Fe^{3+}, Hg^{2+}, Mg^{2+}, K^+, and Zn^{2+}) were prepared in twice distilled water at 2 mM. For fluorescence titration experiments, small aliquots of dsDNA or DOPC solution were successively added into the **MitoMN** (5.0 μM) in buffer solution (10.0 mM Tris-HCl buffer, 100 mM KCl, pH 7.4) and allowed to equilibrate for at least 5 min every time before measured. The analysis of the fluorescence decay profiles was accomplished with decay-analysis software provided by the manufacturer, and the quality of the fit was assessed with the χ^2 value close to unity and with the residuals regularly distributed along the time axis. Data analysis was performed using GraphPad Prism 7.00.

Computational methodologies Compound **MitoMN** was calculations using DFT by Gaussian09 package[2] at B3LYP/6-31g (d, p) level. The effect of the solvent is used with a polarized continuum model (PCM) solvation model in toluene and water.

Molecular docking. The DOPC bilayer system contains 72 1, 2-dioleoyl-sn-glycero-3-phosphocholine (DOPC) molecules, in which the thickness of waters is set to 25 Å, and the **MitoMN** molecule is put in the center of the water layer. The electronic potential of **MitoMN** was calculated using the Gaussian 09

program with the B3LYP function under the 6-311G* basis set.[3] The partial charges of the substrate molecules were derived using the RESP charge fitted with the antechamber module in AMBER 16.[4] The other parameters, including vdW, bond, angle, and torsion terms, were obtained with the antechamber module.[4]. mtDNA preparation: the crystal structure of the mtDNA-ligand complex was downloaded from RCSB Protein Data Bank (mtDNA code: 5JH0). The original ligand and water were removed by PyMOL[5] for docking studies. Ligand (**MitoMN**) preparation: using the optimized compound structure, the partial atomic charges were obtained by restrained electrostatic potential (RESP) [6] calculating with Gaussian 09 package at the level of HF/6-31g*. After that, the docking calculations were performed by the AutoDock 4 suite of programs[7], using a ligand flexible docking approach that allows ligand flexibility. The Lamarckian genetic algorithm[8] was chosen as the search protocol using the default parameters except for the number of GA runs (ga_run = 80) and the maximum number of energy evaluations (ga_num_evals = 2500000). The displaying images were rendered with PyMOL. The hydrophobic contacts were presented by LigPlot$^+$ v.1.4.5 software [9] with hydrophobic-any contact distance 2.9-3.9.

ROS Generation Detection. The ROS generation of **MitoMN** was detected by monitoring the decomposition of 9, 10-anthracenediyl-bis (methylene)dimalonic acid (ABDA) in water and Rose Bengal (RB) was employed as the standard photosensitizer. In these experiments, 5 μL of ABDA stock solution (2 mg/mL) was added into 1 mL of sample solution (the final concentration of ABDA is 10 μg/mL, the final concentration of **MitoMN** or RB is 20.0 μM) under white light irradiation (400–700 nm, 40 mW cm^{-2}). ABDA can react with $^1O_2$ to form the corresponding endoperoxide, leading to an absorption decrease at 378 nm,[10-12] so, the absorbance of ABDA at 378 nm was recorded at different irradiation times to obtain the decay rate of the photosensitizing process. In the ROS generation detection, a standard deviation of 0.1% over 5 repeated experiments. The ROS quantum yield of the photosensitizer (PS) in water (Φ_{PS}) was calculated using the following formula:

$$\Phi_{PS} = \Phi_{RB} \frac{K_{PS} \cdot A_{RB}}{K_{RB} \cdot A_{PS}}$$

where K_{PS} and K_{RB} are the decomposition rate constants of ABDA by the **MitoMN** and RB, respectively. A_{PS} and A_{RB} represent the light absorbed by the **MitoMN** and RB, respectively, which are determined by integration of the areas under the absorption bands in the wavelength range of 400–800 nm. Φ_{RB} is the ROS quantum yield of RB, which is 0.75 in water.

Cytotoxicity Studies. MTT assays were used to assess the cell viability of HeLa and A549 cells. **MitoMN** was dissolved in DMSO (1%, *v/v*), and diluted with fresh media immediately. Cells cultured in 96-well plates were incubated for 24 h to the confluence. After that, cells were incubated with a series of concentrations of **MitoMN** (0 μM-Control, 1.5 μM, 3.1 μM, 6.25 μM, 12.5 μM, 25 μM, 50 μM, 100 μM) for 24 h at 37 °C. And then, the cells were washed with PBS. Then MTT in PBS solution (100 μL, 0.5 mg/mL) was added into each well. After incubation for another 4 h, the supernatant was discarded, and the precipitate was dissolved in DMSO (100 μL) with gentle shaking. The absorbance at 595 nm was measured using a microplate reader (Infinite M200 Pro, Tecan, Switzerland). The cells without any treatment were used as control.

Statistical analysis. All biological experiments were performed at least twice with triplicates in each experiment. Representative results were depicted in this report, and data were presented as means ± SEM with statistical significance.

Supporting Figures and Tables

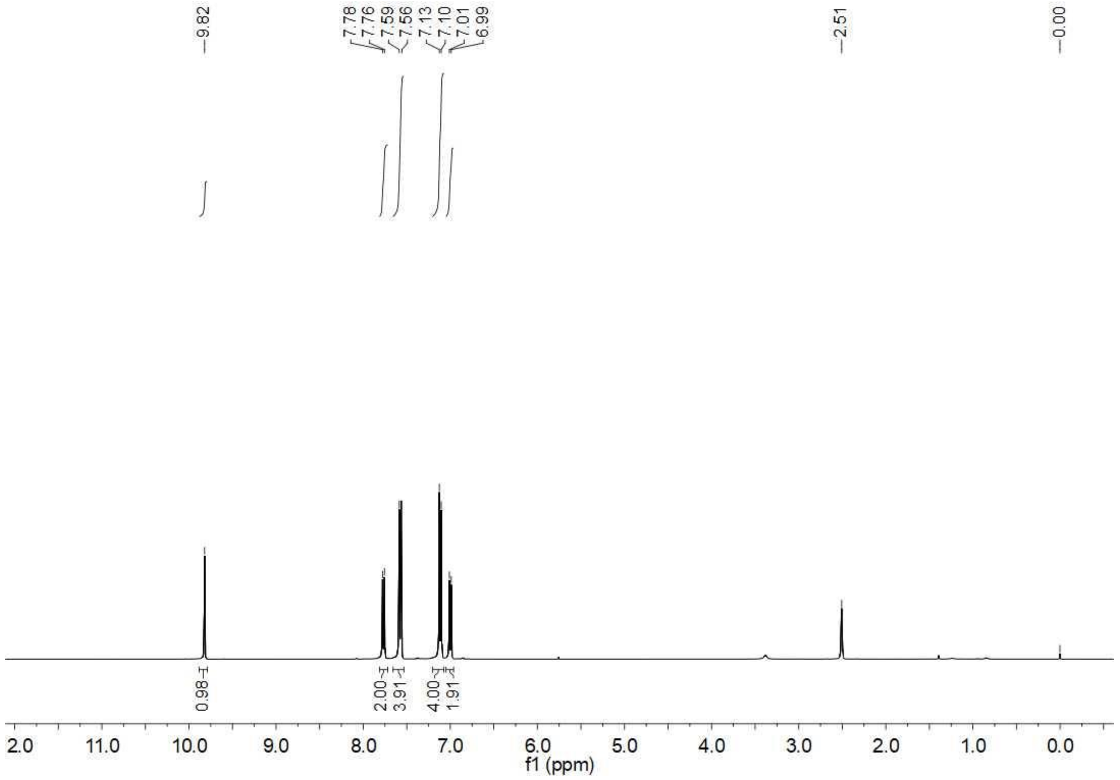

Figure S1. ^{1}H NMR spectrum of **C1**.

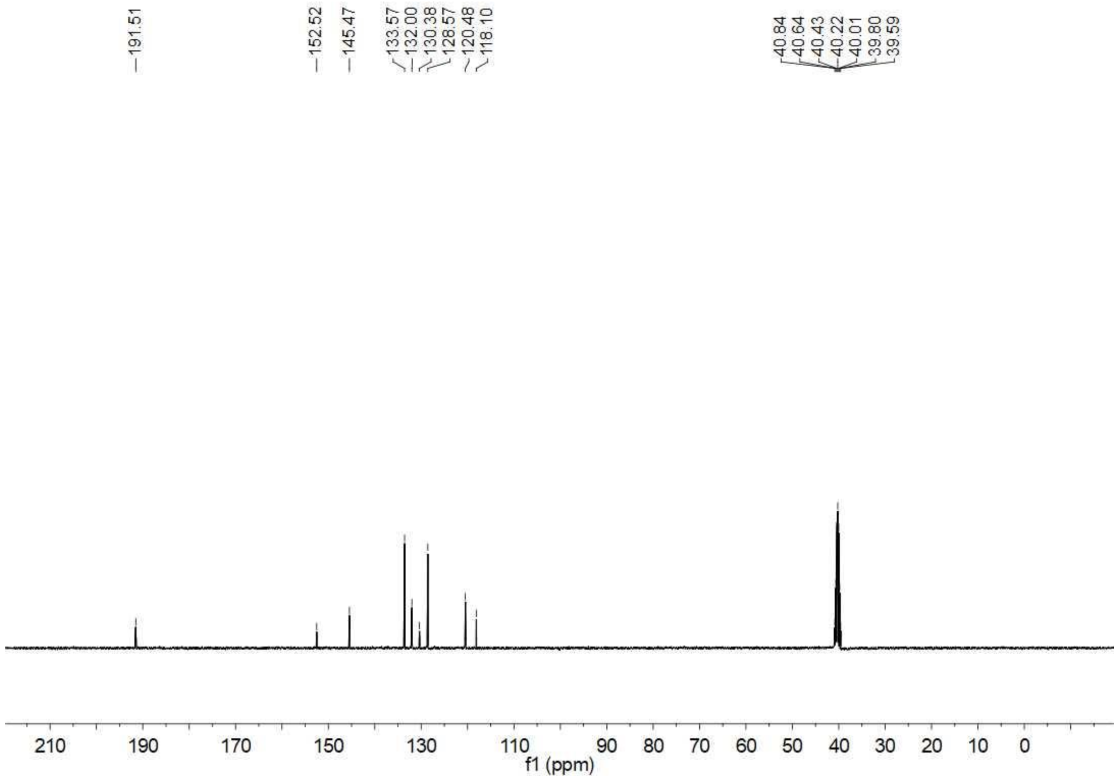

Figure S2. ^{13}C NMR spectrum of **C1**.

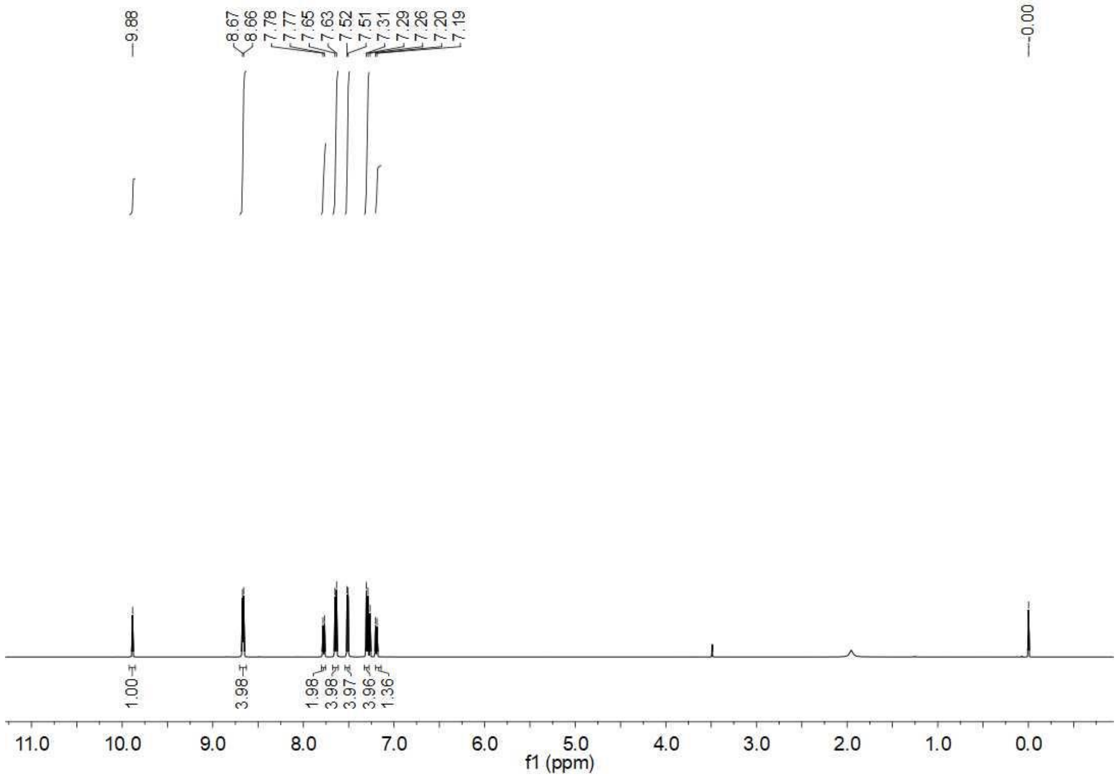

Figure S3. ^{1}H NMR spectrum of **C2**.

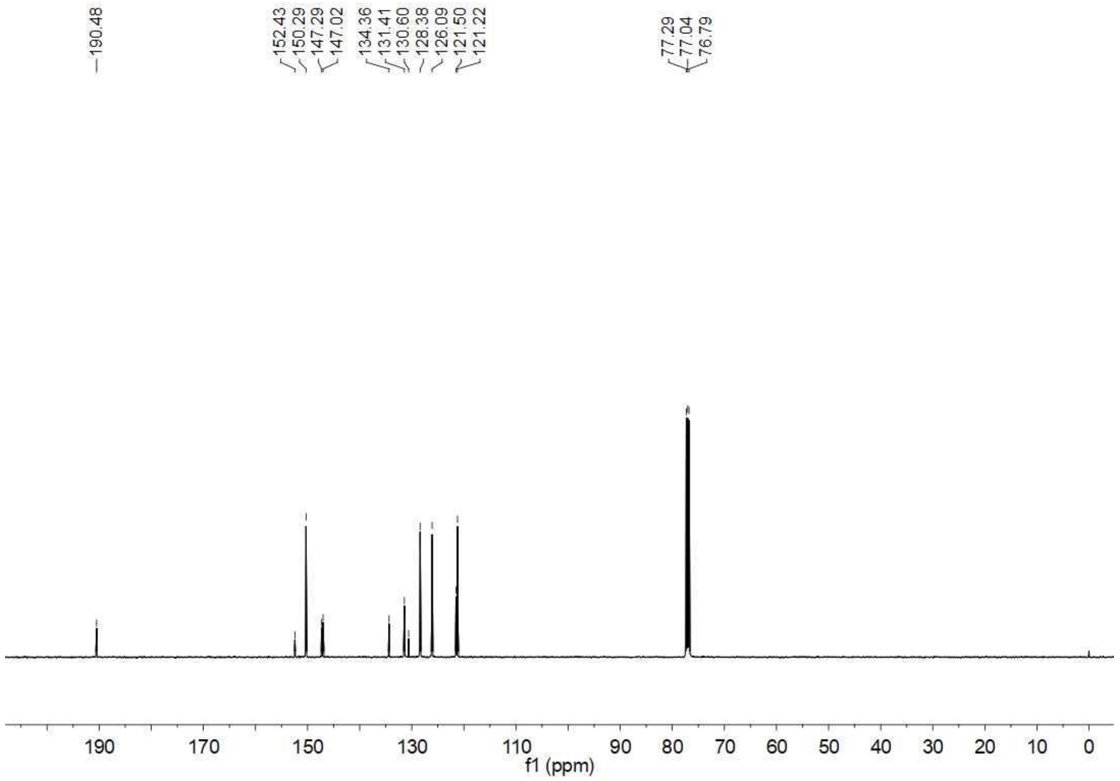

Figure S4. ^{13}C NMR spectrum of **C2**.

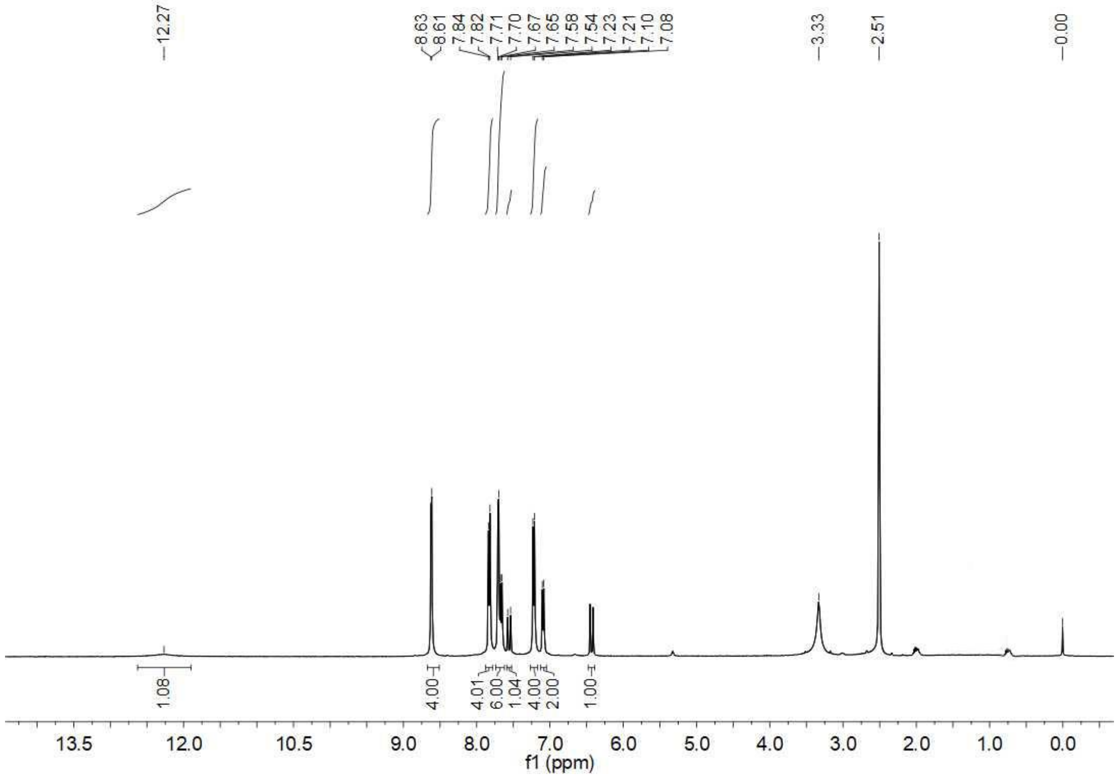

Figure S5. ^{1}H NMR spectrum of **C3**.

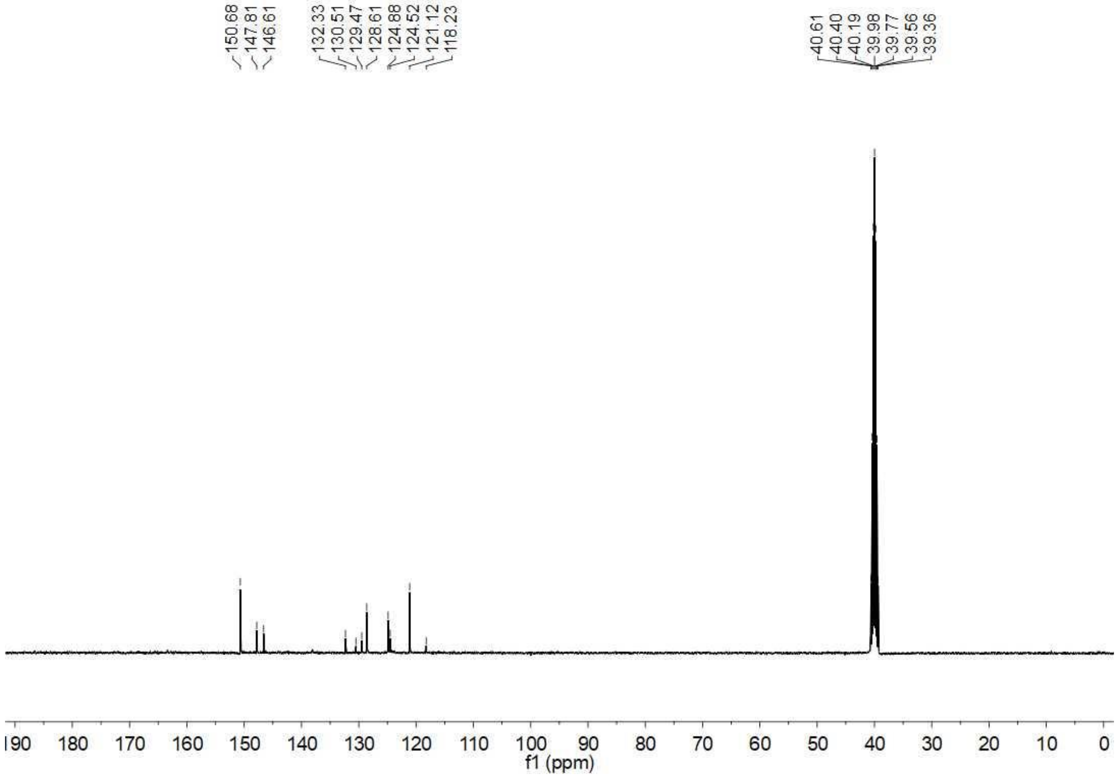

Figure S6. ^{13}C NMR spectrum of **C3**.

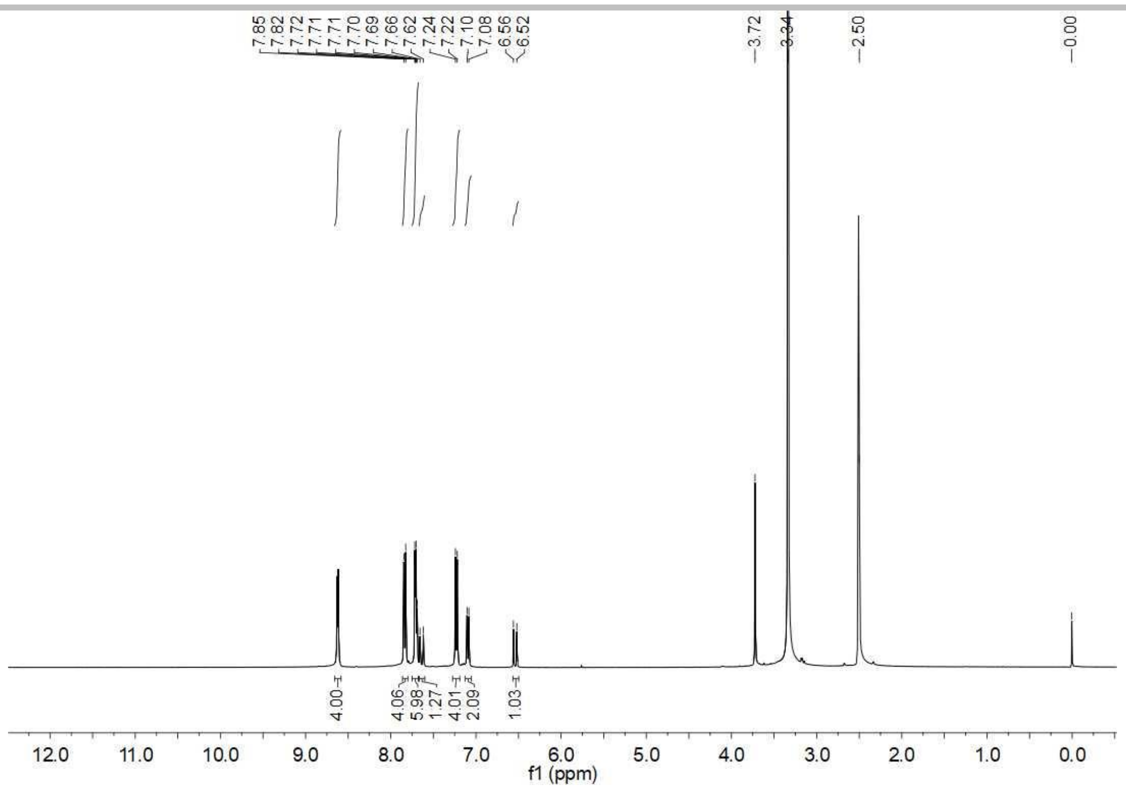

Figure S7. ^{1}H NMR spectrum of **C5.**

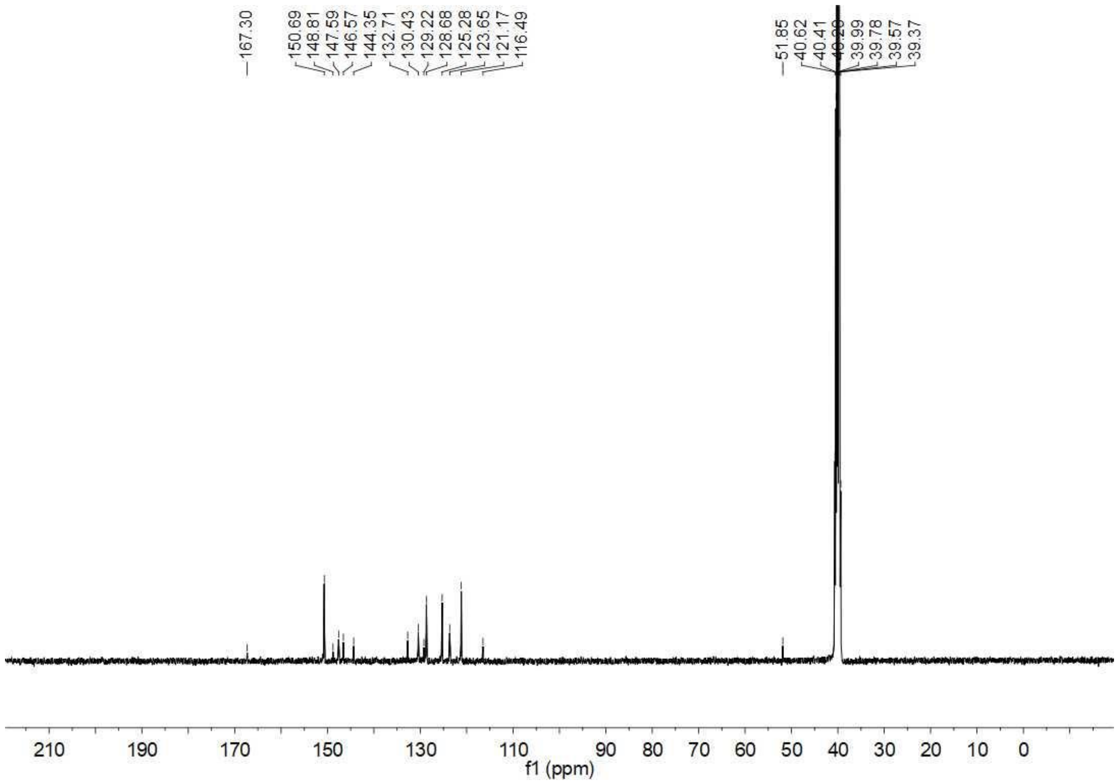

Figure S8. ^{13}C NMR spectrum of **C5.**

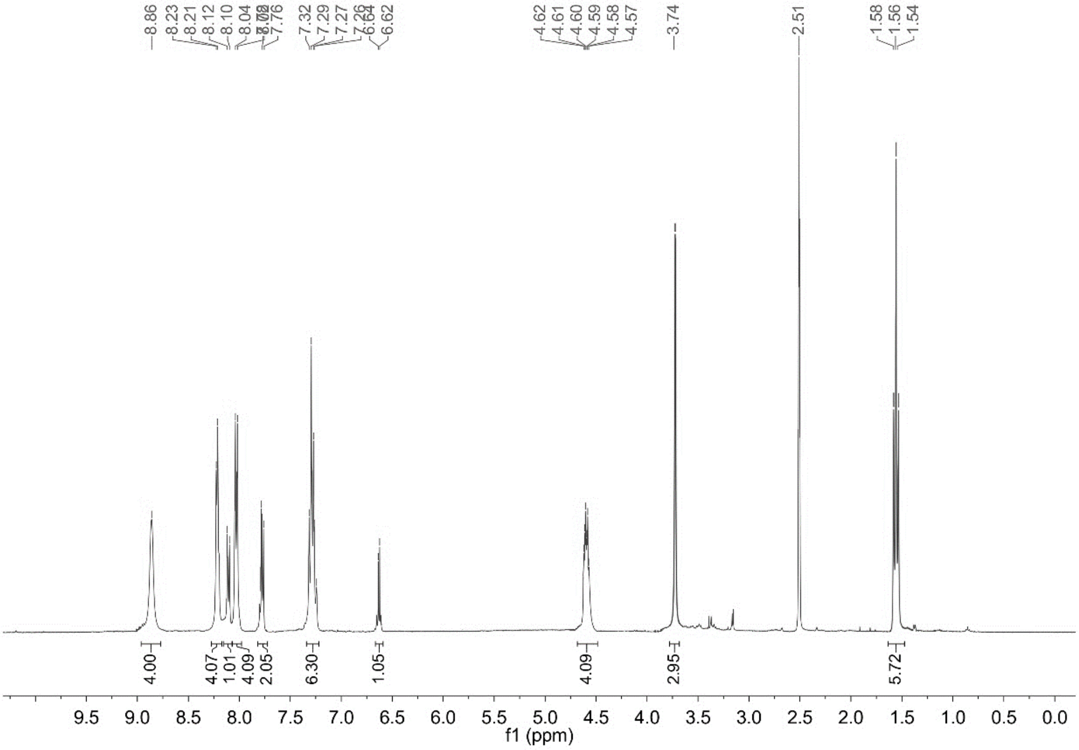

Figure S9. ^{1}H NMR spectrum of **MitoMN.**

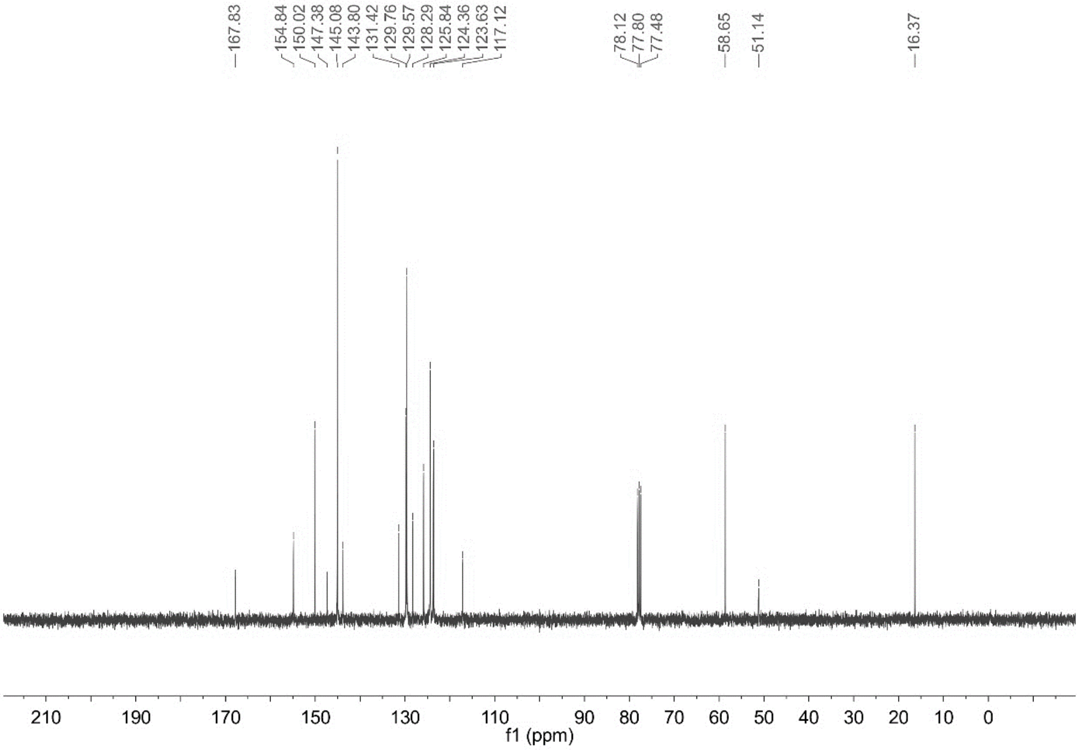

Figure S10. ^{13}C NMR spectrum of **MitoMN.**

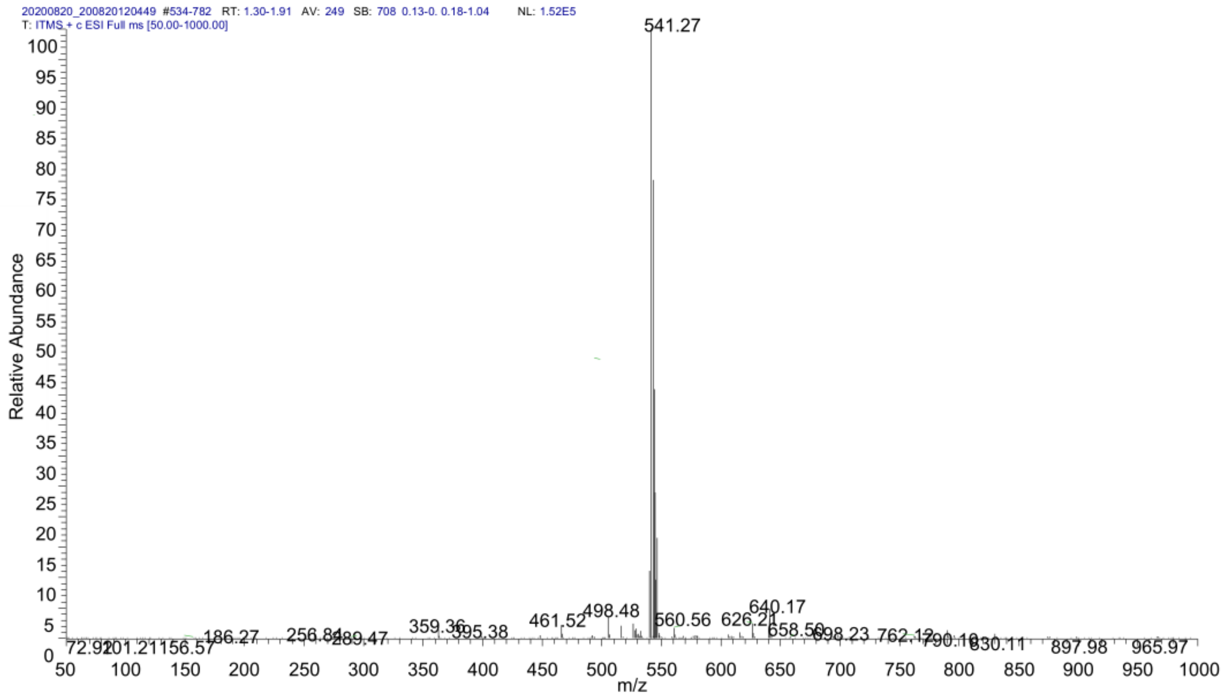

Figure S11. EMS spectrum of **MitoMN.**

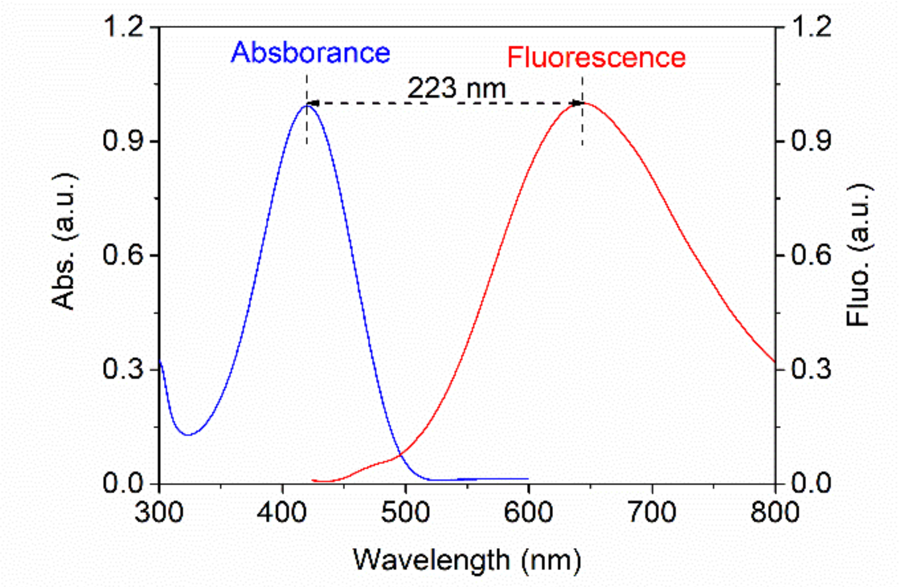

Figure S12. UV-Vis absorption (λ_{abs} = 420 nm, ε = 5.01×10^4 mol^{-1} cm^{-1} L) and fluorescence emission spectra of **MitoMN** (10.0 μM) measured in PBS (pH = 7.4) at 298 K. Due to the appropriate Donor (D)-Acceptor (A) structure, the large Stokes shift (Δλ = 223 nm) of **MitoMN** avoids cross-talk in multicolor imaging to the greatest extent and provides a foundation for large Stokes shift imaging.

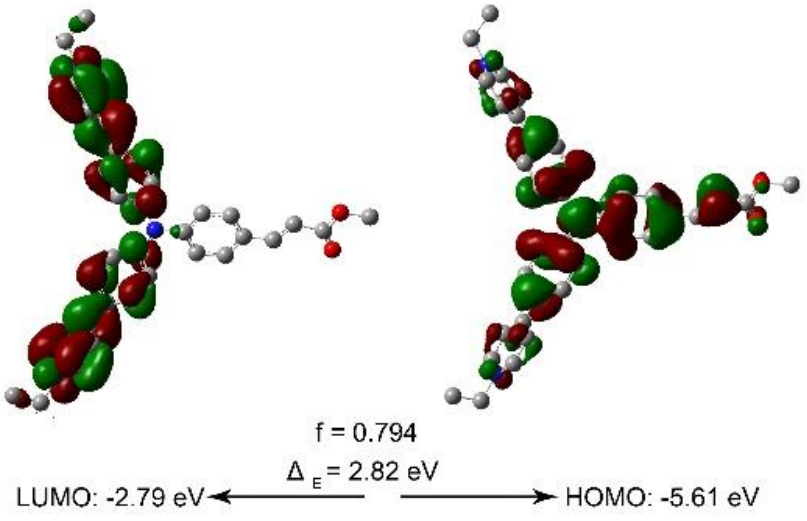

Figure S13. Molecular orbital amplitude plots of the highest occupied molecular orbital (HOMO) and lowest unoccupied molecular orbital (LUMO) energy levels of **MitoMN.** The density functional theory (DFT) calculations indicated that the electron density in the HOMO is delocalized at the triphenylamine segment, while the pyridinium unit dominates the LUMO.

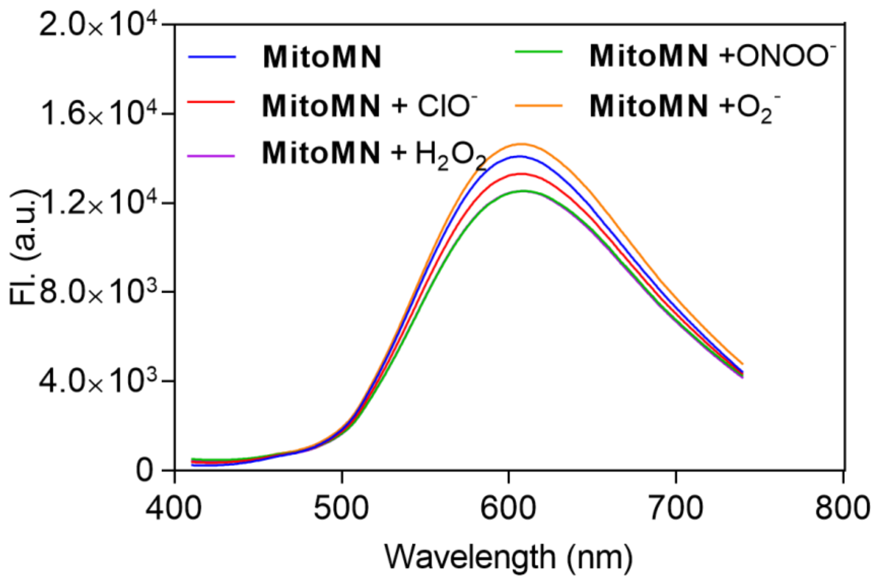

Figure S14. The selectivity of **MitoMN** (10.0 μM) for reactive oxygen species (NOO^-, ClO^-, O_2^-, H_2O_2) at a concentration of 50.0 μM. The fluorescence intensity of **MitoMN** and analytes stabilized for 30 min indicated that the external ROS would not affect the fluorescence of **MitoMN**.

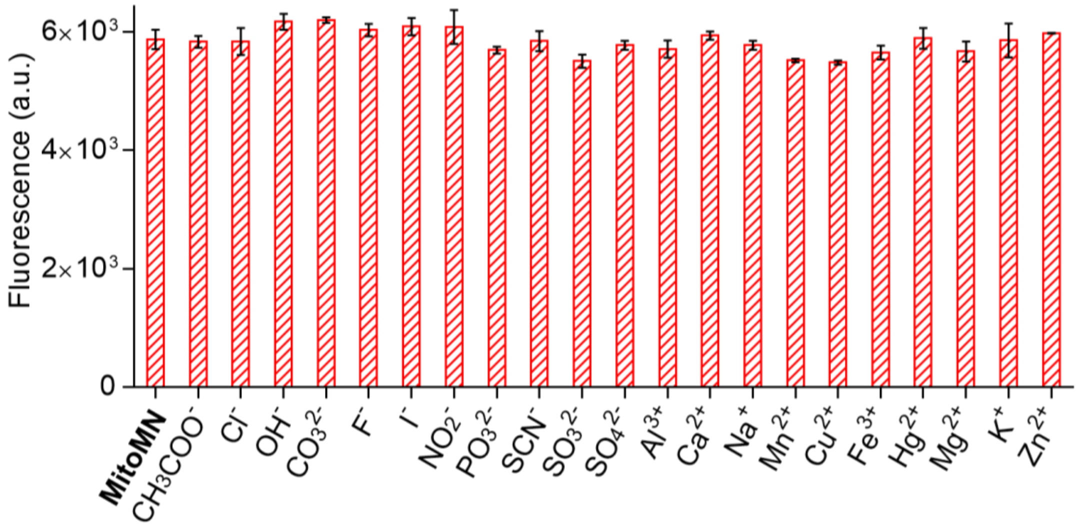

Figure S15. The selectivity of **MitoMN** (10.0 μM) for bioactive anion/cations (50.0 μM). The fluorescence intensity of **MitoMN** and analytes stabilized for 10 min indicated **MitoMN** presented a stable fluorescence emission in the common bioactive anion/cations in buffer solution.

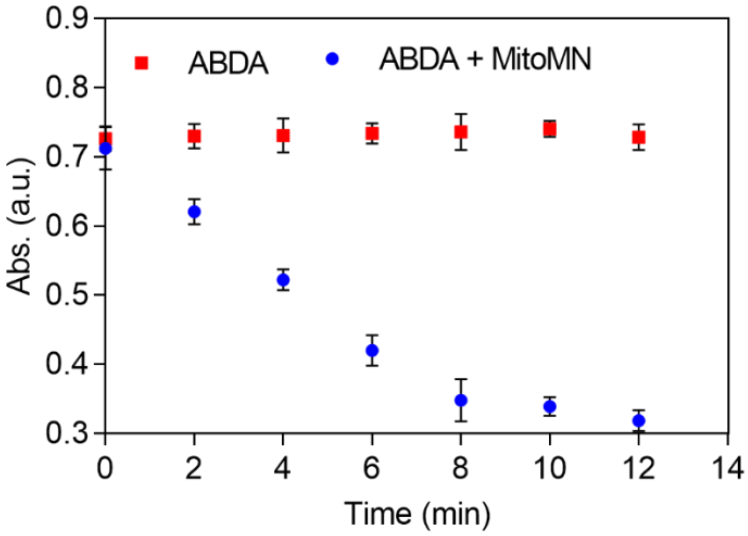

Figure S16. Rate of decay of ABDA sensitized by control and **MitoMN** in PBS, as shown by the decrease in the absorption maxima of ABDA for pH = 7.4, where A_0 and A are the absorbances of ABDA at 378 nm before and after light irradiation. The probe's significant ability to generate ROS (a yield of 73.1±0.1%) provided the ROS overload required for enlargement of mitochondria.

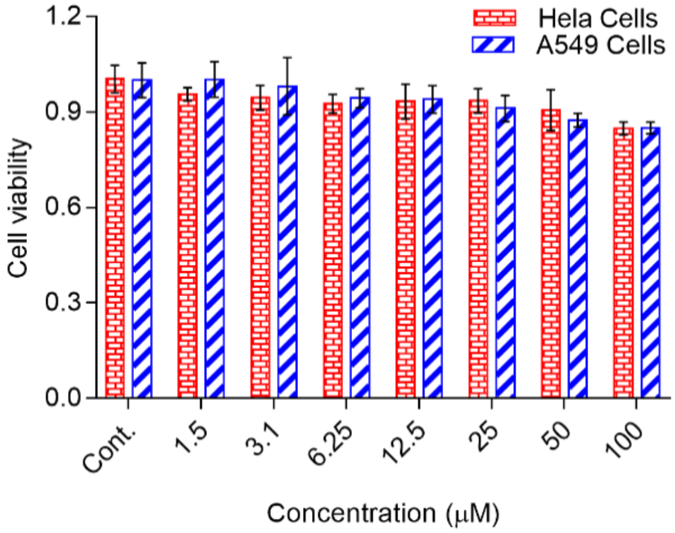

Figure S17. Viability of HeLa/A549 cells treated with the various concentrations of **MitoMN** for 24 h.

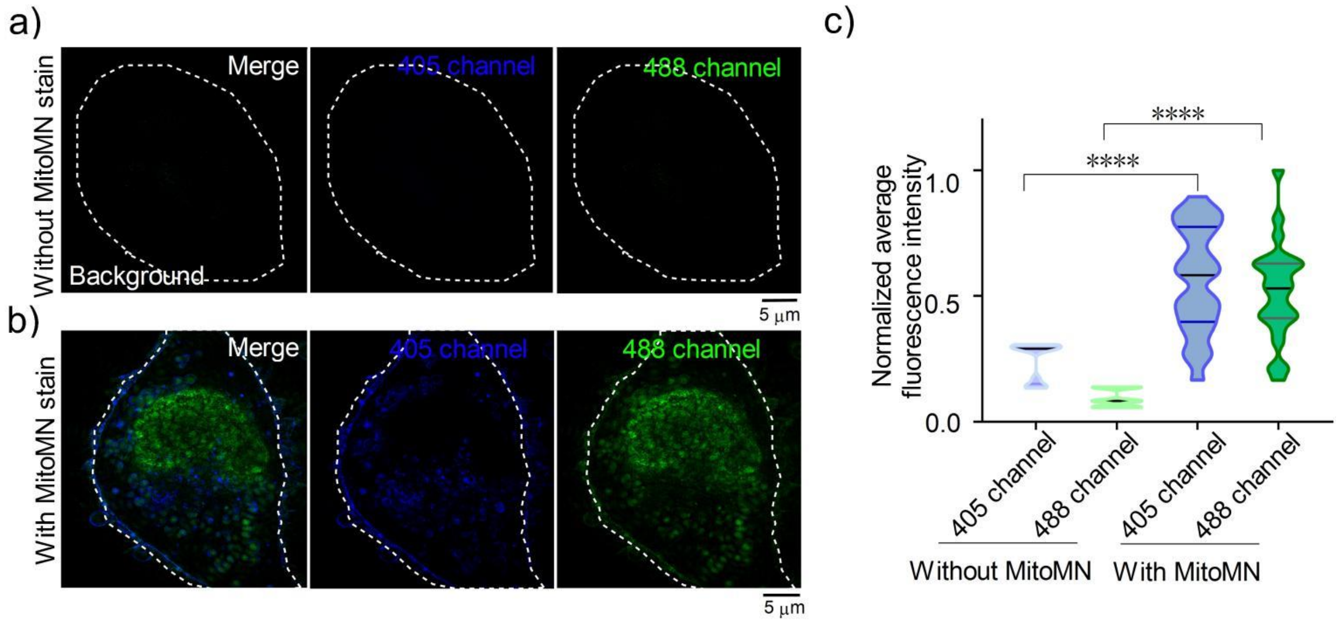

Figure S18. HeLa cells stained without (a) or with (b) **MitoMN** under 405 nm and 488 nm SIM lasers. (c) The normalized average fluorescence intensity. Data were calculated from above 10 fields of view and each experimental condition was performed in triplicate. For SIM imaging, the output powers at the fiber end: 15 mW. The levels of significance were set at n. s. (no significant difference), *P < 0.05, **P < 0.01, ***P < 0.001, and ****P < 0.0001. Data are presented as mean ± SEM (n = 12 fields for without MitoMN group, and n = 24 fields for with MitoMN group).

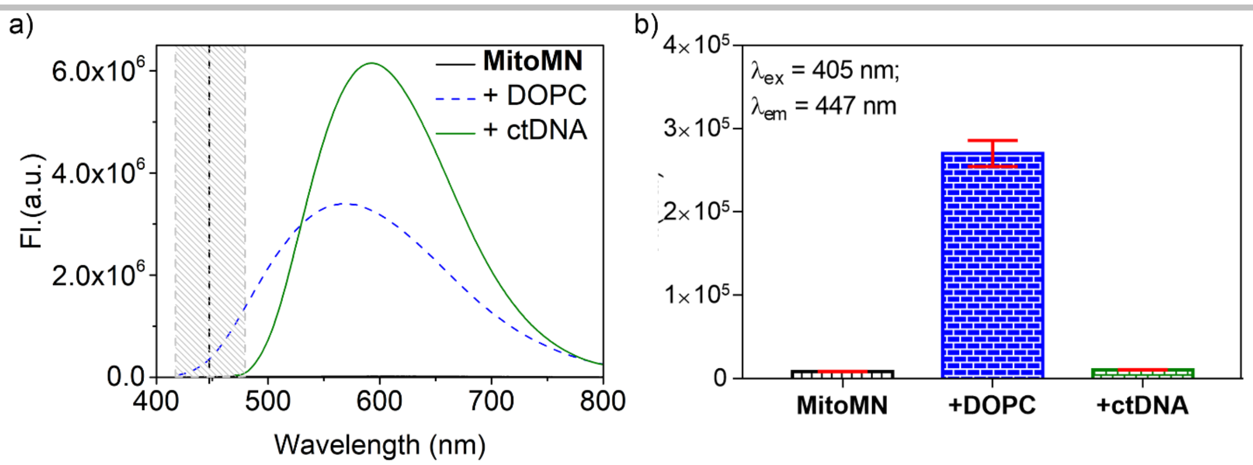

Figure S19. The emission fluorescence spectra of **MitoMN** (10.0 μM) upon DOPC or ctDNA in PBS solution (pH = 7.4), under 405 nm excitation. By comparison, the fluorescence obtained from the **MitoMN**/DOPC mixture solution has a wider emission band and higher fluorescence intensity at 400-500 nm (a). It is worth noting that the difference in fluorescence emission intensity at 447 nm is nearly 26 folds (b), which provide a fluorescence spectral window (417-476 nm) to select lipids from nucleic acids.

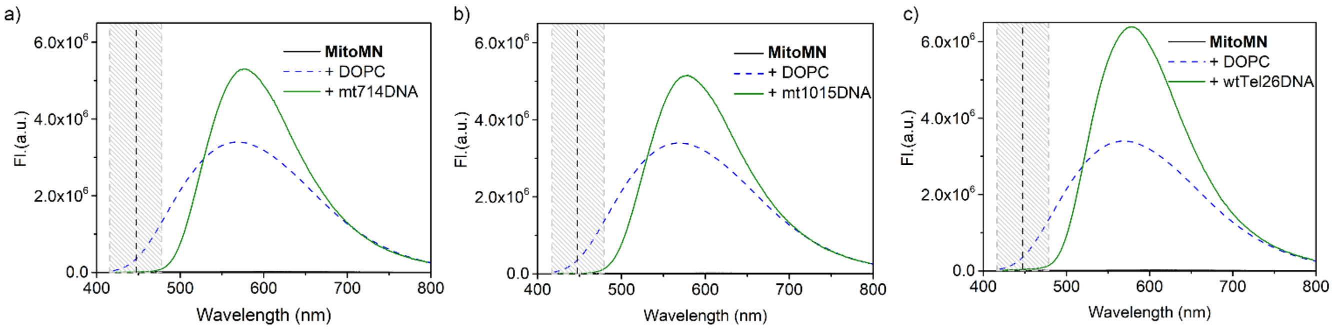

Figure S20. The emission fluorescence spectra of **MitoMN** (10.0 μM) upon DOPC or mitochondrial DNA mt714DNA(a) / mt1015DNA(b) and the G4DNA (wtTel26DNA) (c) in PBS solution (pH = 7.4), under 405 nm excitation.

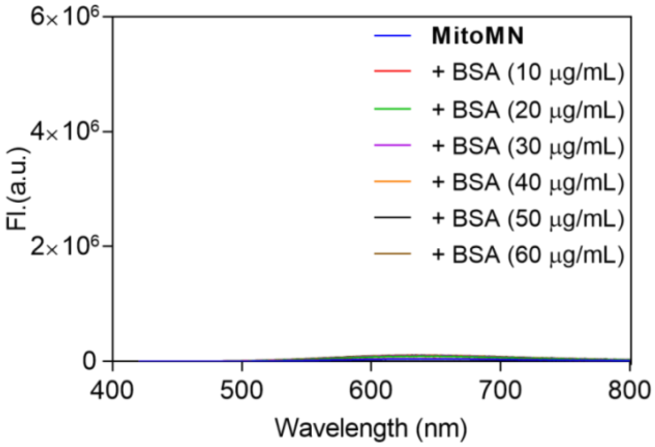

Figure S21. The emission fluorescence spectra of **MitoMN** (10.0 μM) upon protein (Bovine Serum Albumin, BSA) in PBS solution (pH = 7.4), under 405 nm excitation.

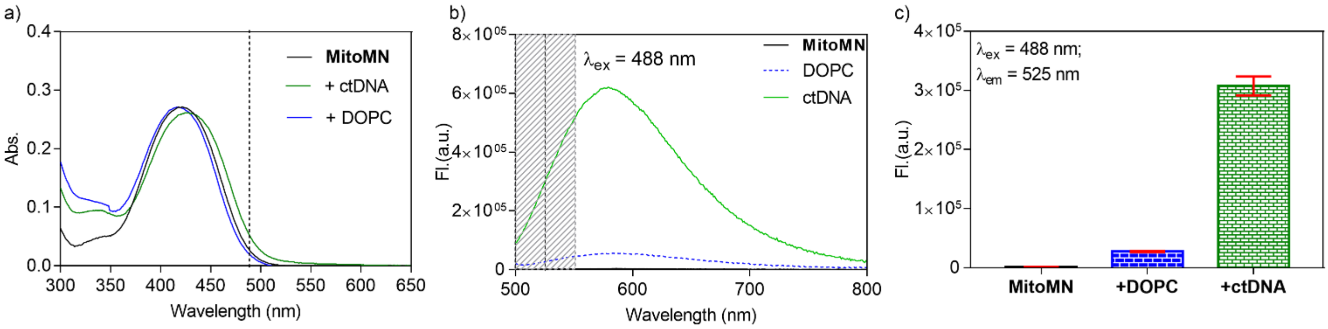

Figure S22. The UV-vis spectra of **MitoMN** (10.0 μM) upon DOPC or ctDNA in PBS solution (pH = 7.4) (a); the emission fluorescence spectra of **MitoMN** (10.0 μM) upon DOPC or ctDNA in PBS solution (pH = 7.4) under 488 nm excitation (b); and the fluorescence intensity of **MitoMN** (10.0 μM) upon DOPC or ctDNA in PBS solution (pH = 7.4) (c).The spectral difference is not only reflected in the fluorescence emission under 405 nm excitation but also in the UV-vis spectrum (c). Compared with the almost unchanged absorption spectrum of **MitoMN**/DOPC mixture, a slight red-shift can be detected in the absorption spectrum of **MitoMN** interact with DNA. Inspired by this change, a longer excitation at 488 nm was used to investigate the fluorescence spectrum changes of **MitoMN** in the interaction of lipids and nucleic acids, respectively. As shown in (b), **MitoMN** shows weaker fluorescence after adding DOPC; however, the fluorescence of **MitoMN** displays greater enhancement after interacted with ctDNA. It is worth noting that the difference in fluorescence emission intensity at 525 nm is nearly 12 folds (c), which can easily meet the requirement of selecting nucleic acids from lipids.

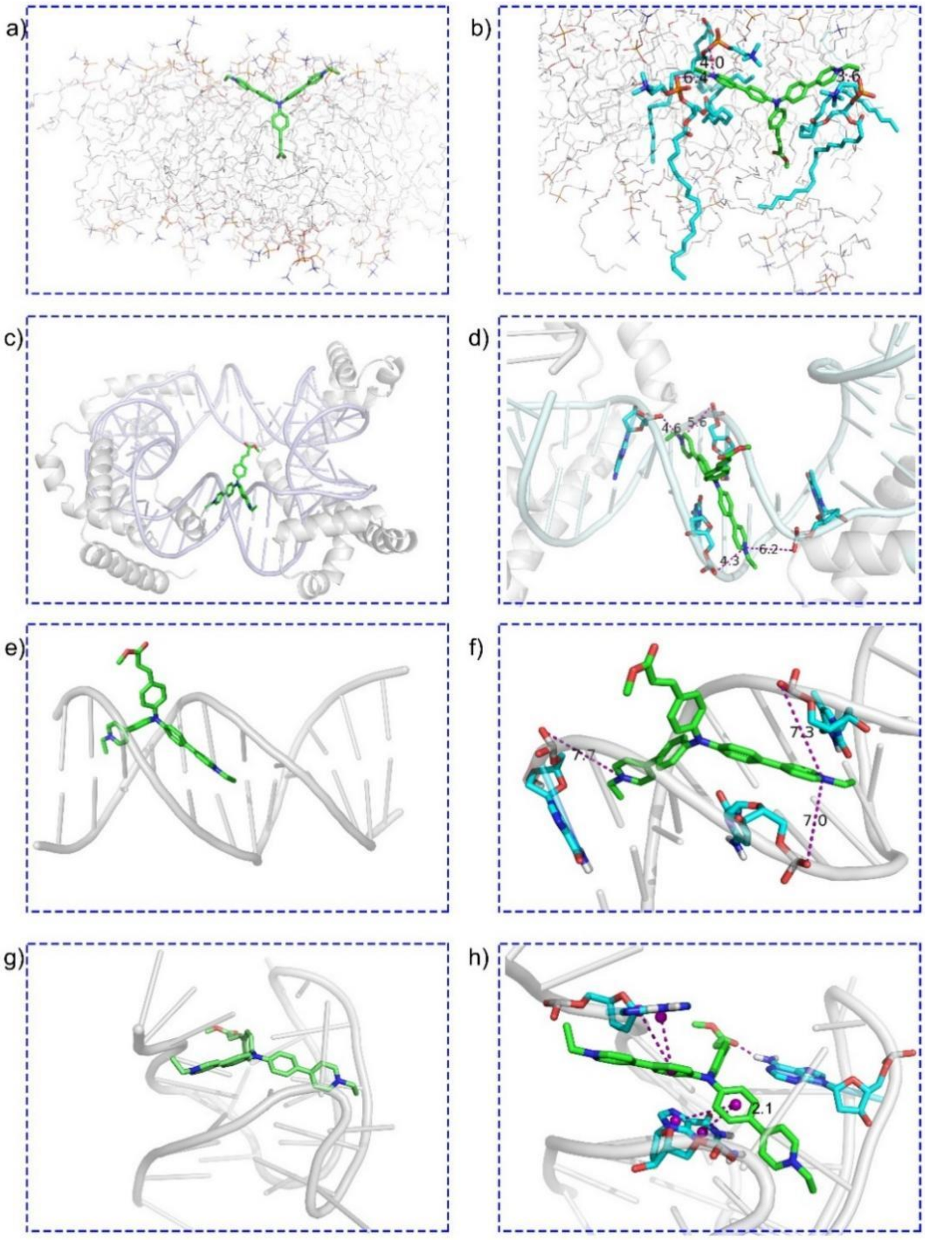

Figure S23. The molecular modeling calculations based on the optimized structure of **MitoMN** and DOPC (a, b), mtDNA (c, d), dsDNA (e, f), and G4-DNA (g, h) structure using AutoDock 4.2 software. The results showed that the positively charged pyridine rings of **MitoMN** interacted with the lipid polar headgroup by strong electrostatic interaction; the steric hindrance of the tripodal **MitoMN** limits its transmembrane process. The combination of **MitoMN** and the simulated membrane is disordered and messy (a, b), which leads to the formation of broader fluorescence emission. However, the sensitive response of **MitoMN** to DNA can be attributed to the **MitoMN** embedded into the hydrophobic minor grooves of DNA (c, d). The regular combination leads the complex to form a sharper and narrower fluorescence emission. This binding form of **MitoMN** is not only reflected in mtDNA but also in the other nucleic acids, such as dsDNA and G4DNA (e, h).

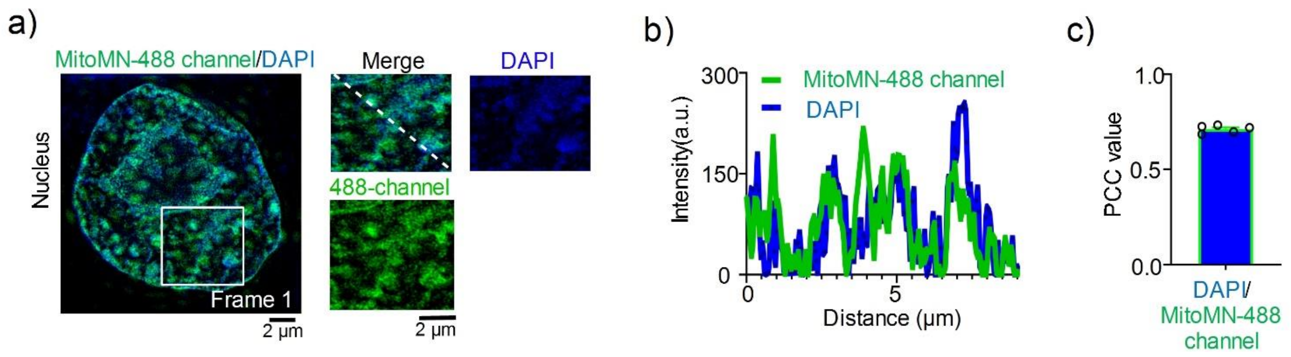

Figure S24. (a) Nucleus stained with DAPI and **MitoMN**, dotted white lines in the merged images indicate where fluorescence intensity profiles shown in (b) were measured; (c) Co-localization value for DAPI and **MitoMN**. **MitoMN** and the commercial nuclear probe, DAPI have been used to co-stain the HeLa cells. Under 488 nm SIM laser conductions, the area in label 1 stained by **MitoMN** was completely located in the DAPI-stained nuclear region (a, enlargement) with a high overlap (b) and co-localization (c). The result showed that **MitoMN** could image the nuclear matrix under 488 nm SIM laser.

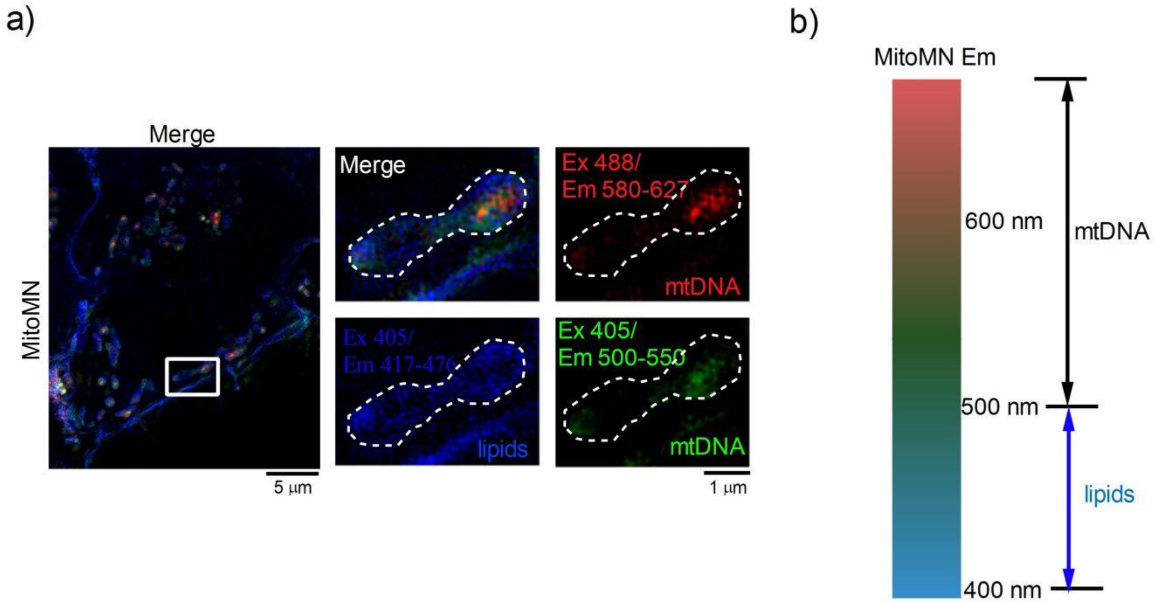

Figure S25. MitoMN labeled mitochondria matrix under different SIM emission and excitation wavelength.

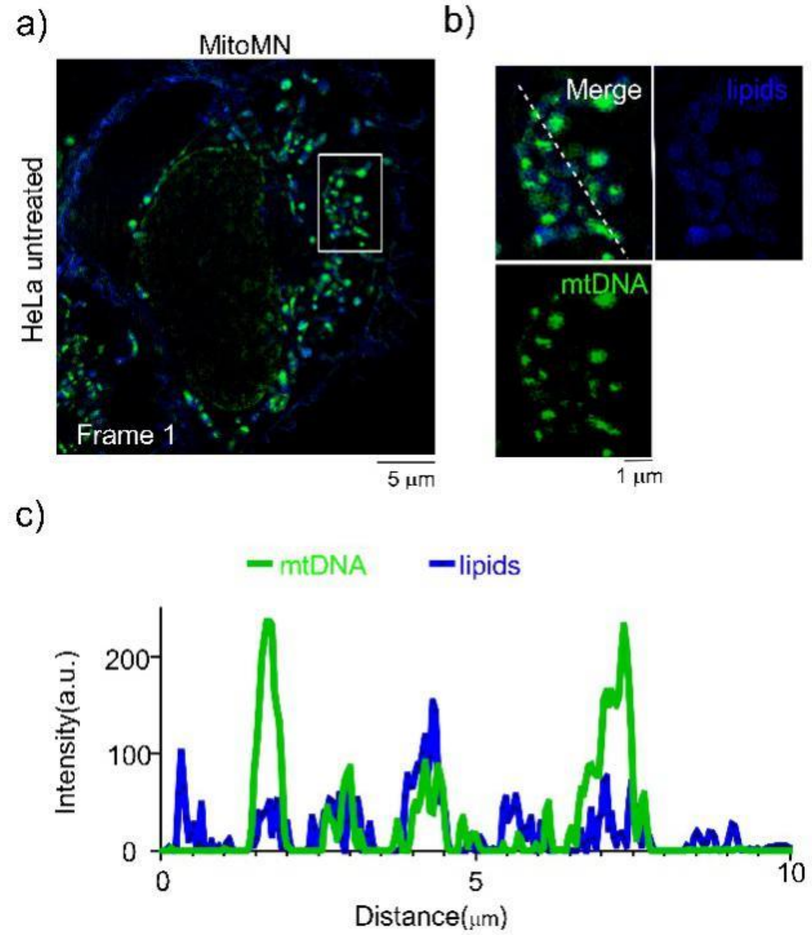

Figure S26. The fluorescence distribution of **MitoMN** stained lipids and **mtDNA** in HeLa untreated cells. (a) Frame 1 image of HeLa cells stained with **MitoMN** under 405 nm and 488 nm SIM lasers. (b) Zoom-in images of white rectangles in (a), white dotted lines indicate region of interest for fluorescence measurement shown in (c), and white circle lines indicate the mitochondria region.

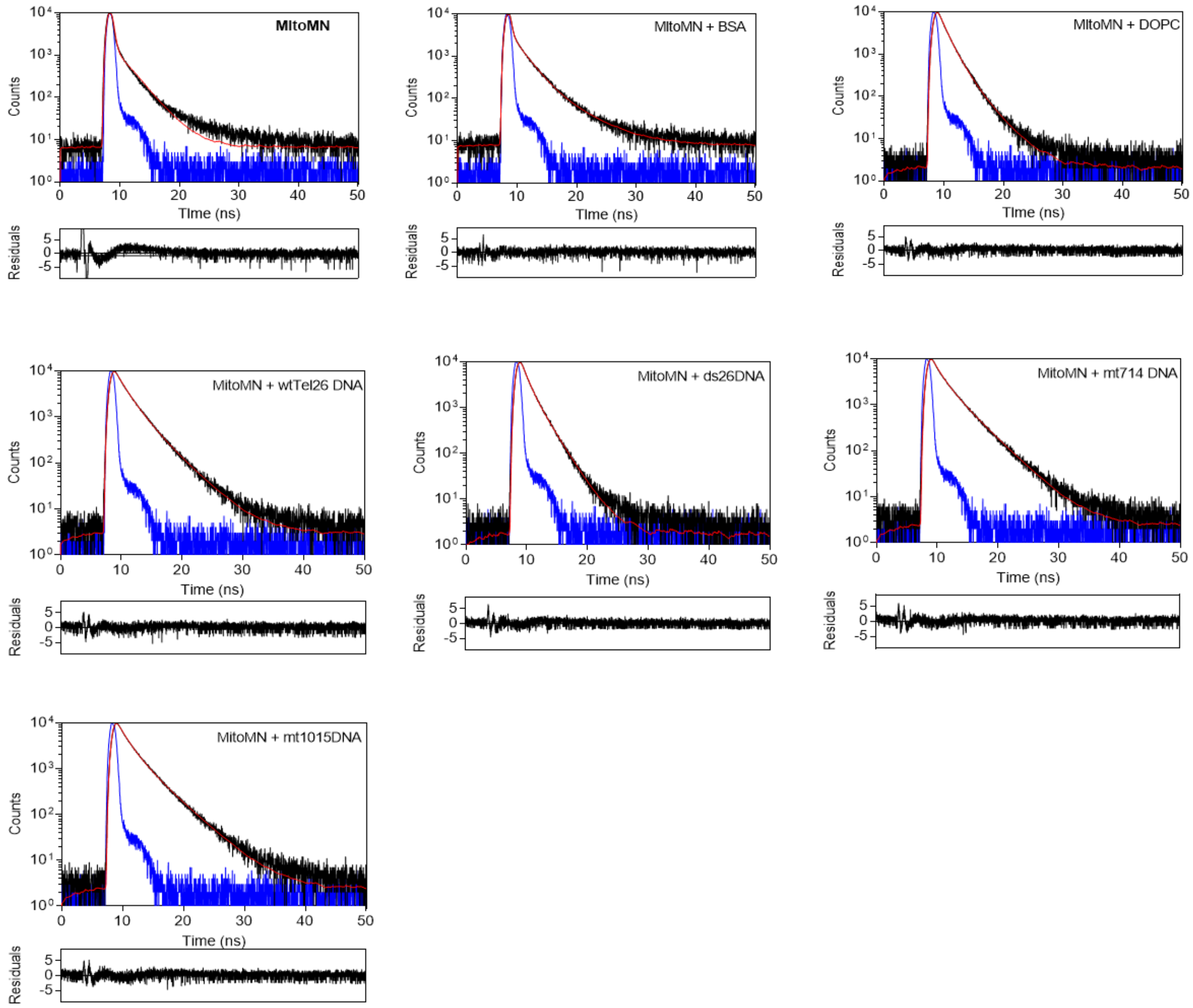

Figure S27. The fluorescence lifetime spectra changes of **MitoMN** binding with BSA, DOPC, and different DNA in PBS, under dark conditions.

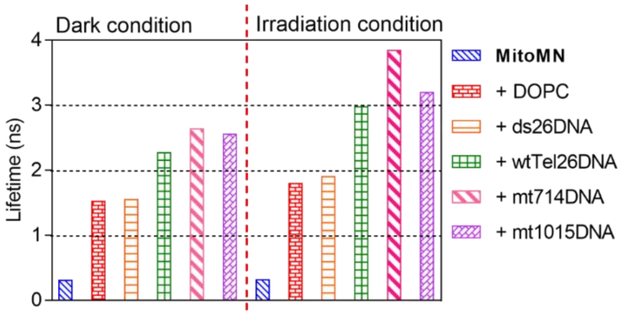

Figure S28. The fluorescence lifetime changes of **MitoMN** binding with BSA, DOPC, and different DNA in PBS under dank or irradiation with 425 nm LED light source.

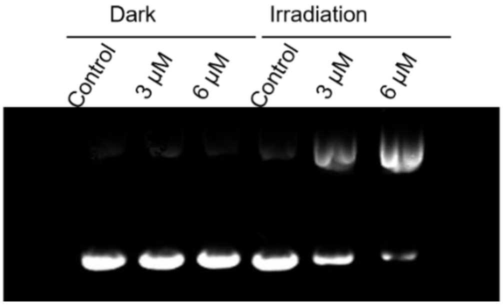

Figure S29. Electrophoresis experiments of DNA photo-cleavage of pBR322 plasmid DNA (0.05 μg/μL) treated with different concentrations (Control (0 μM), 3 μM, 6 μM) of **MitoMN** under dark and light irradiation conditions (425 nm LED light 40 mWcm^{-2}, 10 min) in buffer solution (10.0 mM Tris-HCl, pH = 7.4).

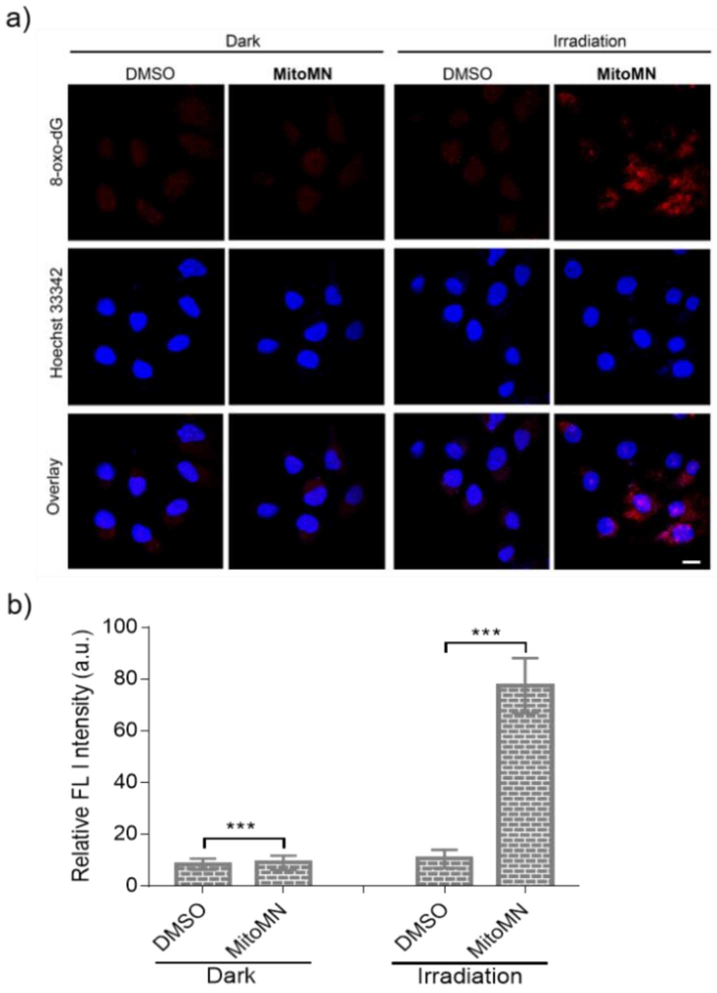

Figure S30. ROS produced by **MitoMN** under irradiation is associated with the accumulation of 8-oxodG. (a) HeLa cells incubated with DMSO under dark or irradiation or incubated with **MitoMN** under the dark or irradiation condition were subjected to immunofluorescence staining with antibodies against 8-oxodG (red), and the nuclei were stained with Hoechst 33342 (blue). Representative confocal imaging the

expression of 8-oxodG in the HeLa cell lines. (b) Quantification of 8-oxodG specific fluorescence in three independent experiments. Relative FL intensity is the ratio between FL intensity in HeLa cells. The levels of significance were set at n.s. (no significant difference), *P < 0.05, **P < 0.01, ***P < 0.001, and ****P < 0.0001. Data are presented as mean ± SEM.

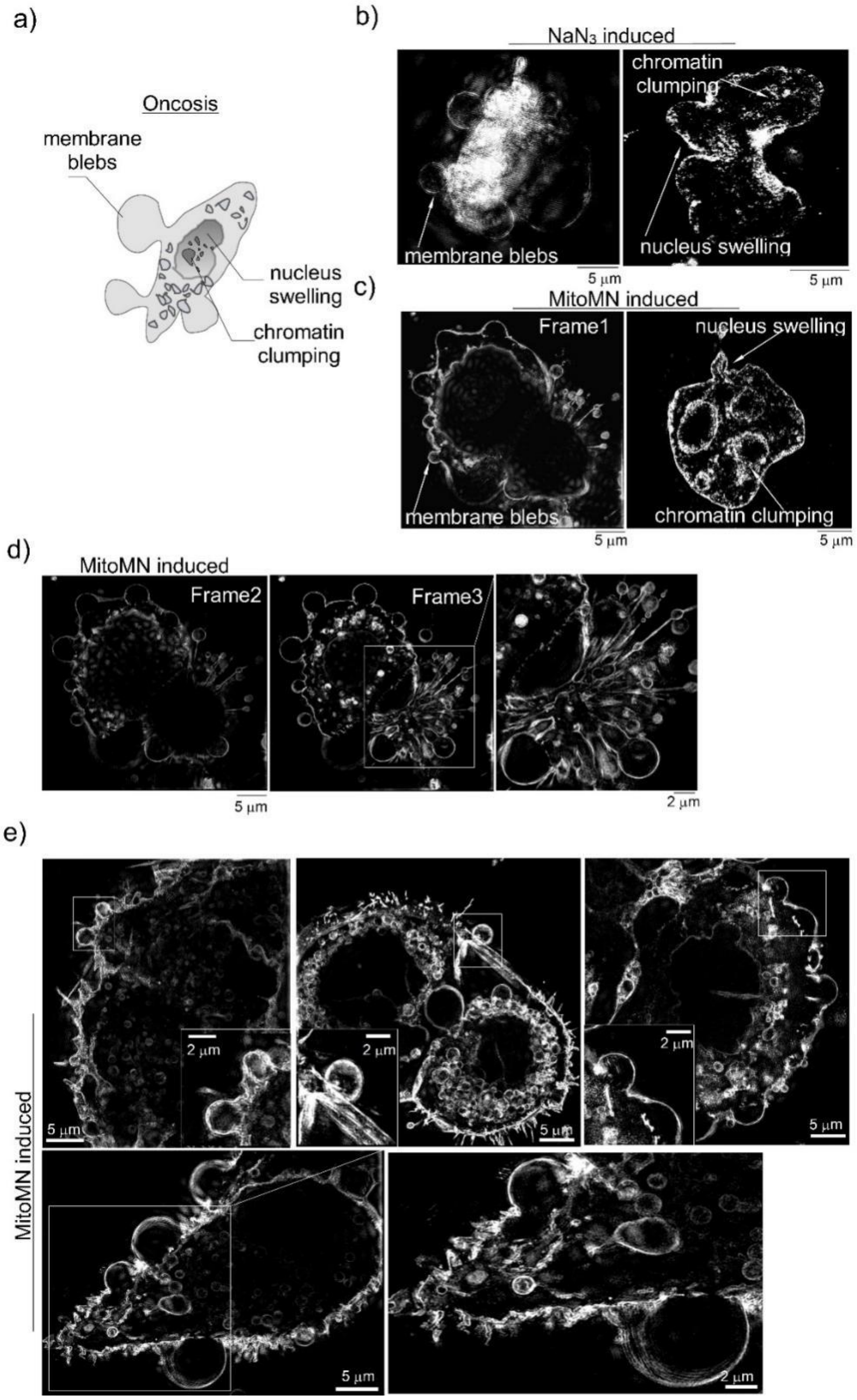

Figure S31. Comparison of nuclei and membrane morphology between MitoMN and NaN_3 stimulation. (a) Schematic representation of oncosis, which is the pre-lethal pathway leading to cell death accompanied

by some typical characteristics, such as, membrane blebs, nucleus swelling, chromatin clumping and so on. After the HeLa cells were incubated with NaN_3 (b) or **MitoMN** under irradiation (c), similar typical events (membrane blebs, nucleus swelling, chromatin clumping) were detected. (d) The HeLa cells incubated with **MitoMN** under irradiation at different frames. (e) The HeLa cells incubated with **MitoMN** under irradiation collected different random fields.

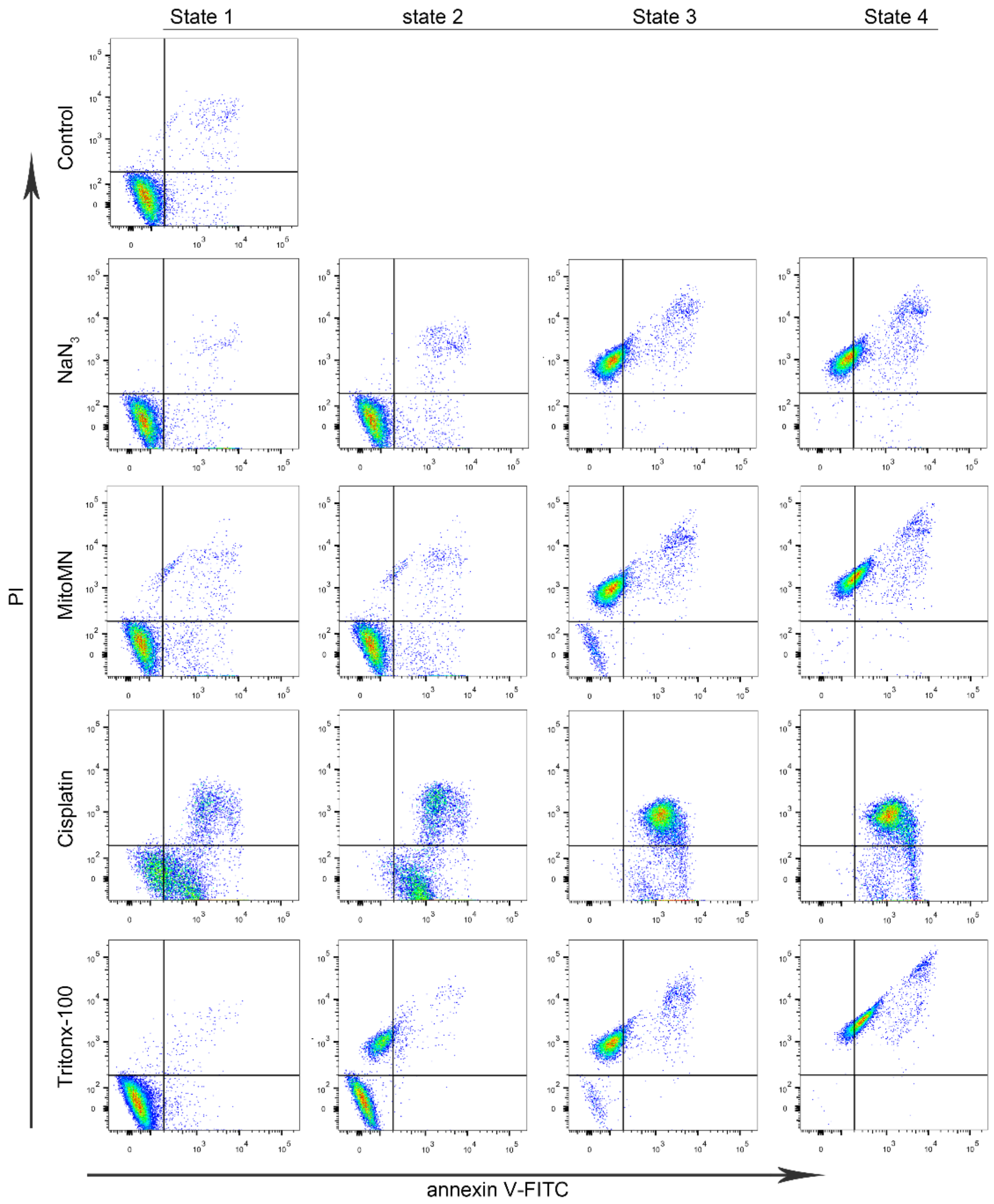

Figure S32 Annexin V-FITC/PI double staining analyzed by flow cytometry. HeLa cells were treated with oncosis inducer NaN_3, **MitoMN**, apoptosis inducer cisplatin, and the necrosis inducer triton-100 at

the indicated concentrations. The fluorescence of Annexin V-FITC (λ_{ex} = 488 nm; λ_{em} = 530 nm) and PI (λ_{ex} = 488 nm; λ_{em} = 630 nm) was analyzed by flow cytometry. The **MitoMN** incubated living cells and after light stimulation showed similar results to that of cells induced by NaN_3. In the early stage, the cells were neither stained by FITC nor PI; while, after being incubated with cisplatin, the cells can be significantly stained by Annexin V-FITC. This apoptosis feature is different from the cell death mode induced by **MitoMN**/NaN_3. For the cells treated with triton-100, although those cells were not stained by Annexin V-FITC, the damage to the cell membrane was direct and continuous, which is accompanied by the process of gradual staining of the nucleus by PI. This necrosis feature also is significantly different from the **MitoMN**/NaN_3 induced cell death mode.

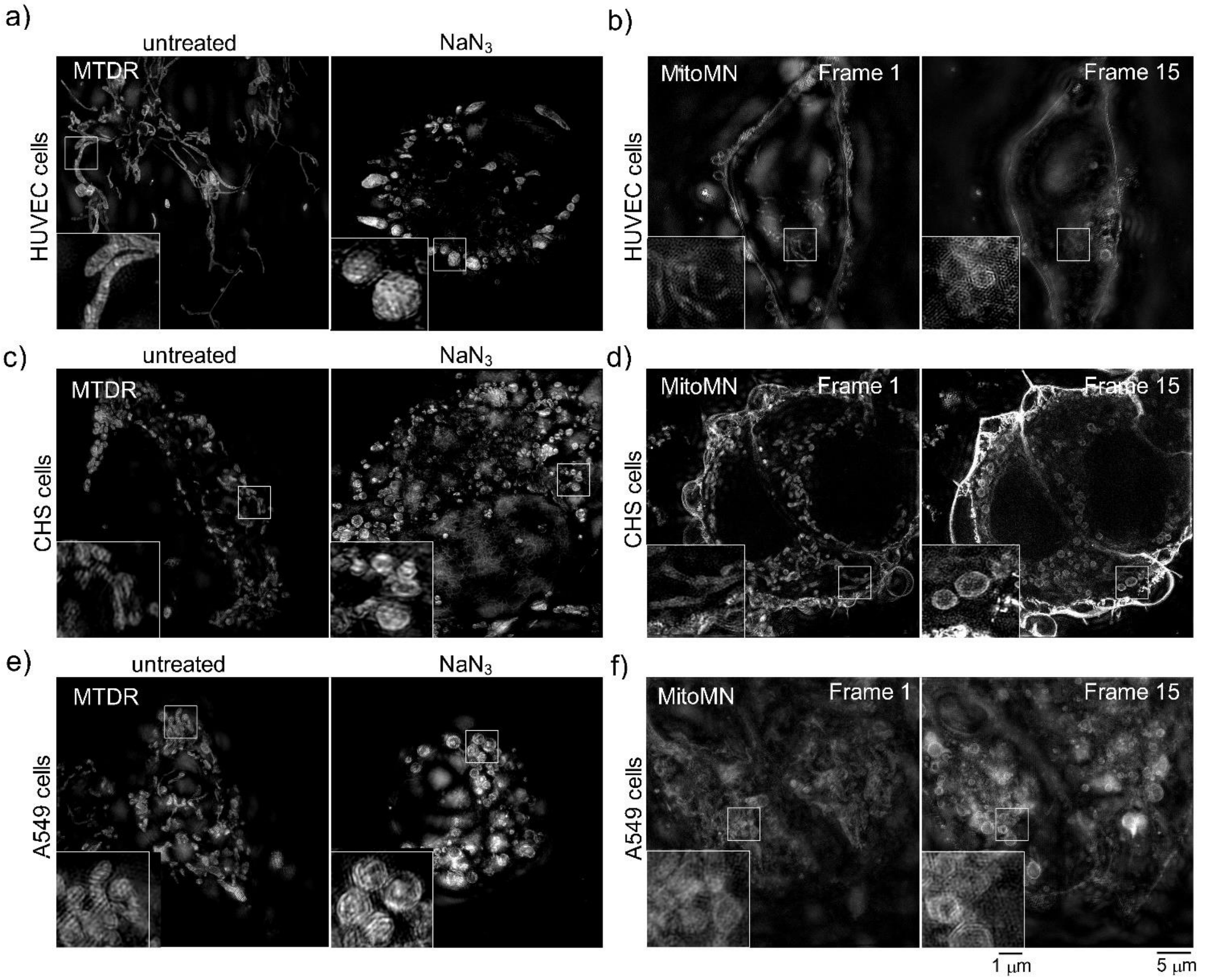

Figure S33. Different cell lines treated with NaN_3 or **MitoMN**. The morphology of mitochondria stained with a commercial mitochondria dye (MTDR, 100 nM) in HUVEC (a), CHS (c) and A549 (e) cells with or without NaN_3 treatment. The morphology of mitochondria stained with **MitoMN** (10.0 μM) in HUVEC (b), CHS (d) and A549 (f) cells.

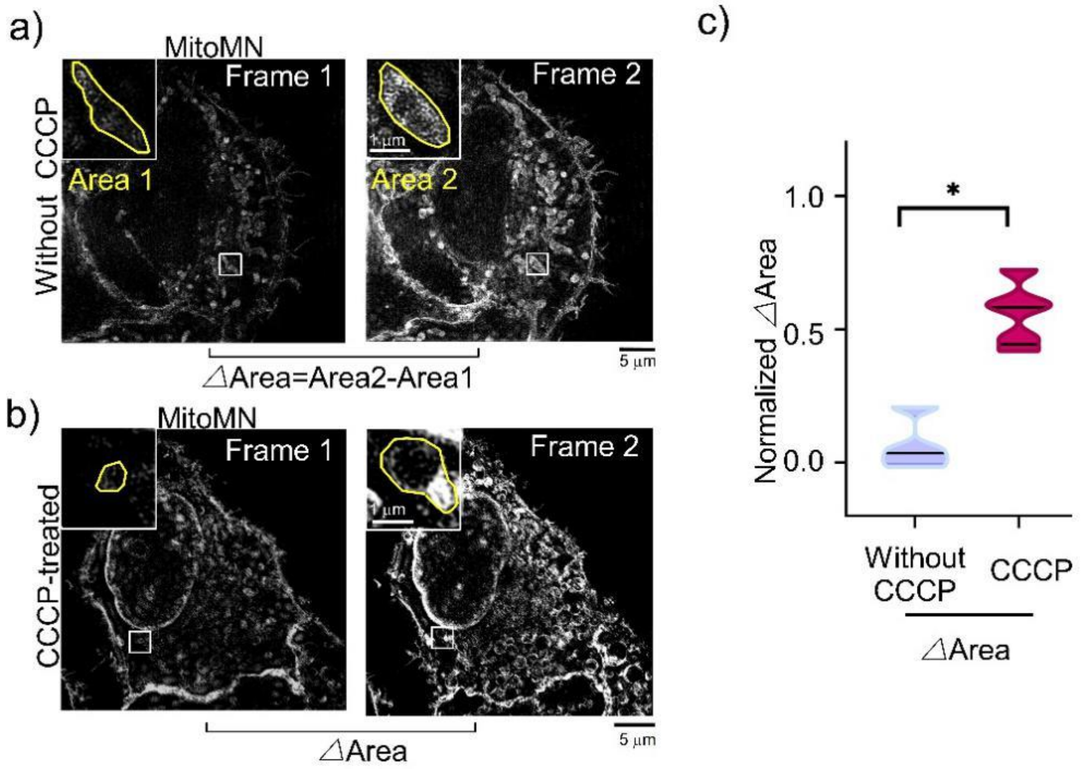

Figure S34. ROS are required for **MitoMN** induced oncosis. The cells with (a) or without (b) CCCP-treatment stained with **MitoMN** under light stimulation; With the same time extension of light stimulation, compared to the untreated cells, the CCCP-treated cells have a larger mitochondrial swelling area (c). The levels of significance were set at n.s. (no significant difference), *P < 0.05, **P < 0.01, ***P < 0.001, and ****P < 0.0001. Data are presented as mean ± SEM (*n* = 4 cells for without CCCP group, and *n* = 5 cells for CCCP group).

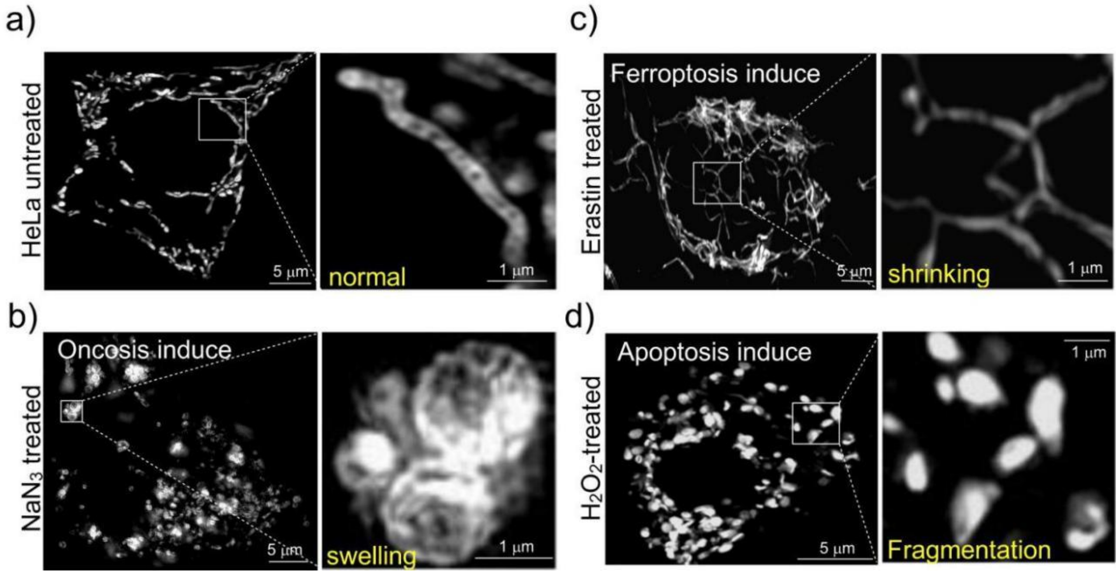

Figure S35. Compared to the untreated HeLa cells (a), NaN_3 induced cell oncosis presented mitochondrial swelling and breakdown (b); the Erastin (ferroptosis inducer) treated cells had noticeable changes in mitochondrial morphology, such as shrinking and the loss of structural integrity (c); and the H_2O_2 induced apoptotic cells showed mitochondrial fragmentation (d).

Table S1. Photophysical properties of MitoMN at room temperature.

	λ_{max}^{abs} (nm)	ε ($10^4 cm^{-1}M^{-1}$)	λ_{max}^{fluo} (nm)	Φ^a
**MitoMN**	420	5.01	651	0.007
**MitoMN** + DOPC	418	5.01	567	0.249
**MitoMN** + DNA	429	4.93	596	0.254

a: Quantum yields were calculated according to literature procedures.[13] Ethanol solution of rhodamine 6G and coumarin 307 were used as the standard, in which the quantum yields of rhodamine 6G and coumarin 307 are 0.94 and 0.56, respectively.

Table S2. The representative docked free energies of the docking models between **MitoMN** and simulated membrane.

**Number of AutoDock clusters** [a]	**Docked free energy range of docked structures (kcal/mol)**	**Cluster rank** [b]	**Docked free energy (kcal/mol)**
		1	-9.07
		2	-8.91
		3	-8.88
		4	-8.73
		5	-8.13
		6	-8.05
		7	-8.02
**26 (80)**	-9.07 to -4.87	8	-7.91
		9	-7.88
		10	-7.86
		11	-7.77
		12	-7.44
		13	-7.15
		14	-7.06
		15	-6.94

[a] Number of GA runs are shown in parentheses.

[b] The cluster rank is the absolute ranking as determined by the docked free energy defined by AutoDock.

Table S3. The representative docked free energies of the docking models between **MitoMN** and mtDNA (PDB: 5JH0).

Number of AutoDock clusters [a]	Docked free energy range of docked structures (kcal/mol)	Cluster rank [b]	Docked free energy (kcal/mol)
		1	-10.58
		2	-10.51
		3	-9.83
		4	-9.80
		5	-9.80
		6	-9.76
		7	-9.72
**23 (80)**	-10.58 to -7.93	8	-9.66
		9	-9.55
		10	-9.53
		11	-9.34
		12	-9.33
		13	-9.30
		14	-9.30
		15	-9.29

[a] Number of GA runs are shown in parentheses.

[b] The cluster rank is the absolute ranking as determined by the docked free energy defined by AutoDock.

Table S4. The representative docked free energies of the docking models between **MitoMN** and dsDNA (PDB: 5ju4).

Number of AutoDock clusters [a]	Docked free energy range of docked structures (kcal/mol)	Cluster rank [b]	Docked free energy (kcal/mol)

		1	-8.35
		2	-8.32
		3	-8.32
		4	-8.30
		5	-8.29
		6	-8.28
		7	-8.28
**2 (80)**	-8.35 to -7.93	8	-8.28
		9	-8.28
		10	-8.28
		11	-8.27
		12	-8.27
		13	-8.26
		14	-8.26
		15	-8.26

[a] Number of GA runs are shown in parentheses.

[b] The cluster rank is the absolute ranking as determined by the docked free energy defined by AutoDock.

Table S5. The representative docked free energies of the docking models between **MitoMN** and G4-DNA (PDB: 6kfj).

**Number of AutoDock clusters** [a]	**Docked free energy range of docked structures (kcal/mol)**	**Cluster rank** [b]	**Docked free energy (kcal/mol)**
**2 (80)**	-6.70 to -6.39<br>Hydrogen bond formed between G4-DNA and **MitoMN**	1	-6.7
		2	-6.66
		3	-6.64
		4	-6.64
		5	-6.63
		6	-6.61

	7	-6.60
	8	-6.59
	9	-6.59
	10	-6.59
	11	-6.58
	12	-6.58
	13	-6.59
	14	-6.57
	15	-6.57

[a] Number of GA runs are shown in parentheses.

[b] The cluster rank is the absolute ranking as determined by the docked free energy defined by AutoDock.

References

[1] Wu, J.W., et al. *Dyes Pigments* **2017**, *136*, 175

[2] Frisch, M. J. et al. *Gaussian 09, Revision D.01.* (Gaussian Inc., Wallingford CT, **2013**).

[3] T. G. Frisch MJ, Schlegel HB, Scuseria GE, Robb MA, Cheeseman JR, Scalmani G, Barone V, Mennucci B, Petersson GA, Nakatsuji H, Caricato M, Li X, Hratchian HP, Izmaylov AF, Bloino J, G. Zheng, Sonnenberg JL, Hada M, Ehara M, Toyota K, Fukuda R, Hasegawa J, M. Ishida TN, Y. Honda, O. Kitao HN, Vreven T, J. A. Montgomery J, Peralta JE, Ogliaro F, Bearpark M, J. J. Heyd EB, Kudin KN, Staroverov VN, Kobayashi R, Normand J, Raghavachari K, Rendell A, Burant JC, Iyengar SS, Tomasi J, Cossi M, Rega N, Millam JM, Klene M, Knox JE, Cross JB, Bakken V, Adamo C, Jaramillo J, Gomperts R, Stratmann RE, Yazyev O, Austin AJ, Cammi R, Pomelli C, Ochterski JW, Martin RL, Morokuma K, Zakrzewski VG, Voth GA, Salvador P, Dannenberg JJ, Dapprich S, A. D. Daniels, Farkas Ö, Foresman JB, Ortiz JV, Cioslowski J, Fox DJ **2009**.

[4] D. Case, R. Betz, D. S. Cerutti, T. Cheatham, T. Darden, R. Duke, T. J. Giese, H. Gohlke, A. Götz, N. Homeyer, S. Izadi, P. Janowski, J. Kaus, A. Kovalenko, T.-S. Lee, S. LeGrand, P. Li, C. Lin, T. Luchko, P. A. Kollman, *Amber 2016, University of California, San Francisco,* **2016**.

[5] DeLano, W. L. *PyMOL(TM) Molecular Graphics System, Version 1.7* (Schrodinger, LLC, **2009**).

[6] C. I. Bayly, P. Cieplak, W. Cornell, P. A. Kollman, *J. Phys. Chem.* **1993**, *97*, 10269.

[7] G. M. Morris, R. Huey, W. Lindstrom, M. F. Sanner, R. K. Belew, D. S. Goodsell, A. J. Olson, *J. Comput. Chem.* **2009**, *30*, 2785.

[8] G. M. Morris, D. S. Goodsell, R. S. Halliday, R. Huey, W. E. Hart, R. K. Belew, A. J. Olson, *J. Comput. Chem.* **1998**, *19*, 1639.

[9] Laskowski, R. *LigPlot⁺ Version 1.4.5* (**2009**).

[10] Ge, J., Lan, M., Zhou, B. et al. *Nat. Commun.* **2014**, *5*, 4596.

[11] H. Chen, Y. Qiu, D. Ding, H. Lin, W. Sun, G. D. Wang, W. Huang, W. Zhang, D. Lee, G. Liu, J. Xie, X. Chen, *Adv. Mater.* **2018**, 30, 1802748.

[12] B. A. Lindig, M. A. J. Rodgers, A. P. Schaap. *J. Am. Chem. Soc.* **1980**, 102, 17, 5590

[13] Crosby G A, Demas J N. J. Chem. Phys. **1971**, 75: 991-1024.
